# The SARS-CoV-2 RNA–protein interactome in infected human cells

**DOI:** 10.1038/s41564-020-00846-z

**Published:** 2020-12-21

**Authors:** Nora Schmidt, Caleb A. Lareau, Hasmik Keshishian, Sabina Ganskih, Cornelius Schneider, Thomas Hennig, Randy Melanson, Simone Werner, Yuanjie Wei, Matthias Zimmer, Jens Ade, Luisa Kirschner, Sebastian Zielinski, Lars Dölken, Eric S. Lander, Neva Caliskan, Utz Fischer, Jörg Vogel, Steven A. Carr, Jochen Bodem, Mathias Munschauer

**Affiliations:** 1 Helmholtz Institute for RNA-based Infection Research, Helmholtz-Center for Infection Research, Würzburg, Germany; 2 School of Medicine, Stanford University, Palo Alto, CA USA; 3 Broad Institute of MIT and Harvard, Cambridge, MA USA; 4 Institute for Molecular Infection Biology, University of Würzburg, Würzburg, Germany; 5 Department of Biochemistry, University of Würzburg, Würzburg, Germany; 6 Institute for Virology and Immunobiology, Julius-Maximilians-University Würzburg, Würzburg, Germany; 7 Department of Biology, MIT, Cambridge, MA USA; 8 Department of Systems Biology, Harvard Medical School, Boston, MA USA; 9 Faculty of Medicine, University of Würzburg, Würzburg, Germany

**Keywords:** SARS-CoV-2, Virology, RNA metabolism, Proteomics

## Abstract

Characterizing the interactions that SARS-CoV-2 viral RNAs make with host cell proteins during infection can improve our understanding of viral RNA functions and the host innate immune response. Using RNA antisense purification and mass spectrometry, we identified up to 104 human proteins that directly and specifically bind to SARS-CoV-2 RNAs in infected human cells. We integrated the SARS-CoV-2 RNA interactome with changes in proteome abundance induced by viral infection and linked interactome proteins to cellular pathways relevant to SARS-CoV-2 infections. We demonstrated by genetic perturbation that cellular nucleic acid-binding protein (CNBP) and La-related protein 1 (LARP1), two of the most strongly enriched viral RNA binders, restrict SARS-CoV-2 replication in infected cells and provide a global map of their direct RNA contact sites. Pharmacological inhibition of three other RNA interactome members, PPIA, ATP1A1, and the ARP2/3 complex, reduced viral replication in two human cell lines. The identification of host dependency factors and defence strategies as presented in this work will improve the design of targeted therapeutics against SARS-CoV-2.

## Abstract (web summary)

Interactions between SARS-CoV-2 viral RNAs and host cell proteins during infection are evaluated to improve our understanding of viral RNA functions and the host innate immune response.

## Main

The rapid spread of a new severe acute respiratory syndrome-related coronavirus (SARS-CoV-2) around the globe has led to a worldwide spike in a SARS-like respiratory illness termed coronavirus disease 2019 (COVID-19)^1^. To date, more than one million lives have been lost due to COVID-19. A detailed understanding of the molecular interactions and perturbations occurring during SARS-CoV-2 infection is required to understand the biology of SARS-CoV-2 and design therapeutic strategies.

SARS-CoV-2 is an enveloped, positive-sense, single-stranded RNA virus that, upon infection of a host cell, deploys a ‘translation-ready’ RNA molecule, which uses the protein synthesis machinery of the host to express a set of viral proteins crucial for replication^2^. Replication of the full-length viral genome and transcription of subgenomic RNAs both involve the synthesis of negative-strand RNA intermediates^3^. In common with other RNA viruses, SARS-CoV-2 is dependent on effectively engaging host cell factors such as regulators of RNA stability, processing, localization and translation to facilitate replication and production of progeny. The host cell, on the other hand, must detect the pathogen and activate appropriate innate immune response pathways to restrict virus infection^4^.

Studies on SARS-CoV-2-infected human cells to date have focused on characterizing expression or modification changes in the host cell transcriptome^5,6^ or proteome^7–9^. Further, interactions between recombinant viral proteins and host proteins have been identified in uninfected cells^10,11^. Mapping of the interactions between viral and host proteins has revealed cellular pathways relevant to productive infection^12^. However, these studies cannot reveal how viral RNA is regulated during infection or how host cell RNA metabolism is remodelled to enable virus replication^13^.

We sought to obtain an unbiased and quantitative picture of the cellular proteins that directly bind to SARS-CoV-2 RNAs in infected human cells. Recent RNA capture and quantitative mass spectrometry (MS) approaches^14–17^ applied ultraviolet (UV) crosslinking to create covalent bonds between RNA molecules and the proteins they directly interact with. Unlike chemical crosslinking, UV irradiation does not stabilize protein–protein or RNA–RNA interactions, making it a preferable choice for dissecting direct RNA–protein interactions^18,19^. RNA antisense purification and quantitative mass spectrometry (RAP–MS) combines UV crosslinking with a highly denaturing purification procedure and is ideally suited to capture and identify only those proteins that bind directly to SARS-CoV-2 RNAs^14,15^.

## Results

### Capturing SARS-CoV-2 RNAs in infected human cells

To purify SARS-CoV-2 RNAs and the complement of directly crosslinked cellular proteins from infected human cells, we designed a pool of biotinylated DNA oligonucleotides antisense to the positive-sense SARS-CoV-2 RNA and its subgenomic messenger RNAs. As a cellular system, we selected the human liver cell line Huh7, which is naturally permissive to both SARS-CoV-1 and SARS-CoV-2 replication^20,21^. SARS-CoV-2 preferentially infects cells in the respiratory tract, but infection of multiple organs, including the liver, has been reported^22^.

**Fig. 1:**
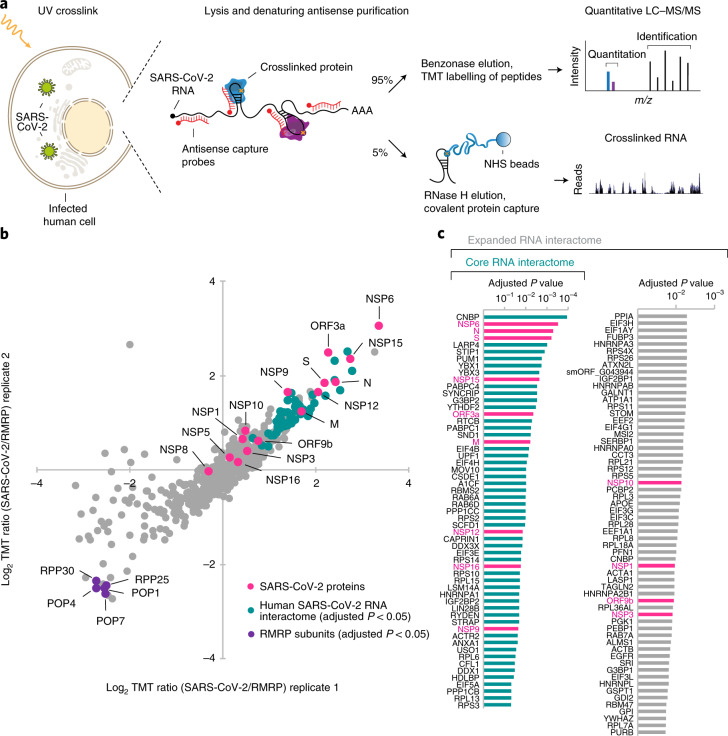
RNA–protein interactome of SARS-CoV-2 in infected human cells. **a**, Outline of the RAP–MS method to identify proteins bound to SARS-CoV-2 RNA and their crosslinked RNA sequences. **b**, Quantification of SARS-CoV-2 RNA-interacting proteins relative to RMRP-interacting proteins. The scatter plot of log_2_-transformed TMT ratios from two biological replicates is shown. The grey dots represent all proteins detected with two or more unique peptides. **c**, Proteins enriched in SARS-CoV-2 RNA purifications (Supplementary Table 1). Left: core SARS-CoV-2 RNA interactome (adjusted *P* < 0.05). Left and right: expanded SARS-CoV-2 RNA interactome. Significantly enriched proteins are highlighted in teal; SARS-CoV-2-encoded proteins are highlighted in magenta. Adjusted *P* value: two-tailed moderated *t*-test.

**Extended Data Fig. 1:**
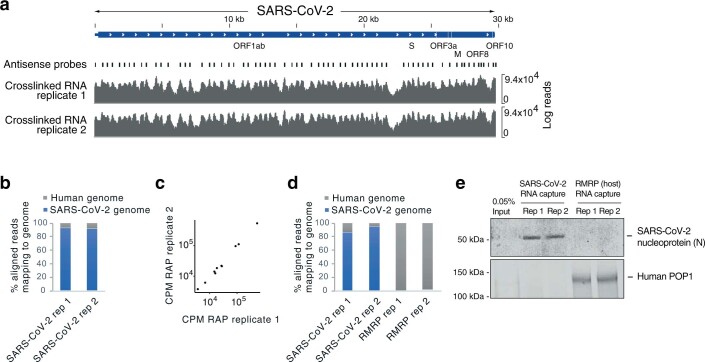
Capturing SARS-CoV-2 RNAs and bound proteins with RAP-MS. **a**, Alignment of protein-crosslinked RNA fragments to the SARS-CoV-2 genome following RNA antisense purification of SARS-CoV-2 RNAs from infected Huh7 cells. Two replicate experiments are shown. **b**, Fraction of crosslinked RNA fragments mapping to the human or SARS-CoV-2 genomes in pilot RAP-MS experiments. **c**, Correlation plot for two replicate RAP experiments. CPM values for SARS-CoV-2 genes are shown. CPM: counts per million. **d**, As in **b**, but for full-scale SARS-CoV-2 RNA RAP-MS and RMRP RAP-MS experiments. **e**, Western blot of two replicate SARS-CoV-2 RNA and RMRP RAP-MS experiments. Indicated antibodies were used for protein detection. Source data

To test if our pool of antisense capture probes was suitable for the purification of SARS-CoV-2 RNAs from infected Huh7 cells, we performed RAP–MS 24 h after infection when viral replication levels were high^21^. We implemented a covalent protein capture step after the release of SARS-CoV-2 RNA-bound proteins, which enabled us to identify RNA sequences crosslinked to purified proteins (Fig. 1a and Methods). Protein-crosslinked RNA fragments mapped to the entire length of the viral genome with near-complete sequence coverage, indicating that interactions across all viral RNA regions were captured (Extended Data Fig. 1a). Sequencing reads originating from SARS-CoV-2 RNA made up 93 and 92% of all mapped reads in 2 highly correlated replicate experiments (*r* = 0.994; Extended Data Fig. 1b,c).

To identify proteins that specifically interact with SARS-CoV-2 RNAs as opposed to non-specific background proteins, we compared the protein content of SARS-CoV-2 RNA purifications to that of an unrelated control ribonucleoprotein complex of known composition. As the control, we used the endogenously expressed human ribonuclease mitochondrial RNA processing (RMRP) RNA and purified both SARS-CoV-2 RNA and RMRP from infected Huh7 cells. RMRP was selected for several reasons: (1) RMRP interacts with approximately ten well-known proteins that serve as an internal control^15,23^; (2) RMRP is not translated; and (3) RMRP does not globally bind to mRNA. Hence, RMRP-binding proteins are distinct from the group of proteins expected to bind to SARS-CoV-2 RNAs, making it an ideal control for the discovery of unknown interactors. Further, the purification of SARS-CoV-2 RNA and RMRP from infected cells avoids biases resulting from widespread changes in the host cell proteome induced by viral infection.

On average, approximately 90% of all crosslinked RNA fragments originated from the SARS-CoV-2 genome in SARS-CoV-2 RNA purifications, while more than 99% of crosslinked RNA fragments from RMRP purifications mapped to the human genome (Extended Data Fig. 1d). Western blot analysis confirmed the specific capture of SARS-CoV-2 nucleocapsid protein only in SARS-CoV-2-purified samples (Extended Data Fig. 1e). The RMRP component POP1 was detected only in RMRP purifications. Together, these experiments verify the high specificity of our approach for capturing the desired RNAs and the proteins that directly bind to them.

### An atlas of SARS-CoV-2 RNA–protein interactions in human cells

Next, we subjected proteins purified with RMRP and SARS-CoV-2 RNAs to tandem mass tag (TMT) labelling and relative quantification by liquid chromatography coupled with tandem mass spectrometry (LC–MS/MS). In two replicate experiments, we identified 699 proteins, of which 583 were detected with 2 or more unique peptides (Supplementary Table 1 and Methods). As shown in Fig. 1b, we found five known RMRP components among the ten most significantly enriched proteins in RMRP purifications.

Next, we analysed proteins enriched in SARS-CoV-2 RNA purifications and found 15 SARS-CoV-2 proteins, 6 of which were among the 20 most significantly enriched proteins (Fig. 1b,c). In addition to 5 viral proteins translated from distinct open reading frames (ORFs), 10 of the 16 non-structural proteins (NSPs), which are derived from a precursor polyprotein^24^, were detected by RAP–MS.

As expected, the SARS-CoV-2 nucleocapsid protein, which binds the viral RNA, was one of the two most significantly enriched viral proteins, followed by several known viral RNA binders, such as the endoribonuclease NSP15 (ref. ^25^), the RNA-dependent RNA polymerase (RdRP) NSP12 (ref. ^26^), the methyltransferase NSP16 (ref. ^27^), the RNA-binding protein NSP9 (ref. ^28^), the capping factor NSP10 (ref. ^27^), the primase NSP8 (ref. ^26^), the 5′-UTR binder NSP1 (ref. ^29^) and the multifunctional protein NSP3 (ref. ^30^). Remarkably, NSP3 and the most strongly enriched protein in our data, NSP6, were required for the formation of double-membrane vesicles^31^ and both proteins are candidate constituents of a molecular pore complex involved in the export of RNA from coronavirus double-membrane vesicles^32^. We also found ORF3a, which binds the 5′-end of the SARS-CoV-1 genome^33^, as well as ORF9b and the S and M proteins among strongly enriched candidates. While M is known to interact with the nucleocapsid protein, a model for genomic RNA packaging further suggests a possible RNA-binding function for M^34^. An RNA-binding activity of S was not previously reported. While S covers the surface of the viral envelope, it has a transmembrane domain and an intracellular tail^35^, making it conceivable that S may indeed contact viral RNA.

### Discovery of 104 human proteins that bind SARS-CoV-2 RNA

We next focused on the human proteins enriched in SARS-CoV-2 RNA purifications. We identified 276 proteins with a positive log_2_ fold change. Of these, 57 were significantly enriched (adjusted *P* < 0.05, two-tailed *t*-test), which we subsequently defined as the set of core SARS-CoV-2 RNA interacting proteins (Fig. 1c). Additionally, we also defined an expanded SARS-CoV-2 RNA interactome using a relaxed false discovery rate (FDR) of less than 20% (Fig. 1c).

**Fig. 2:**
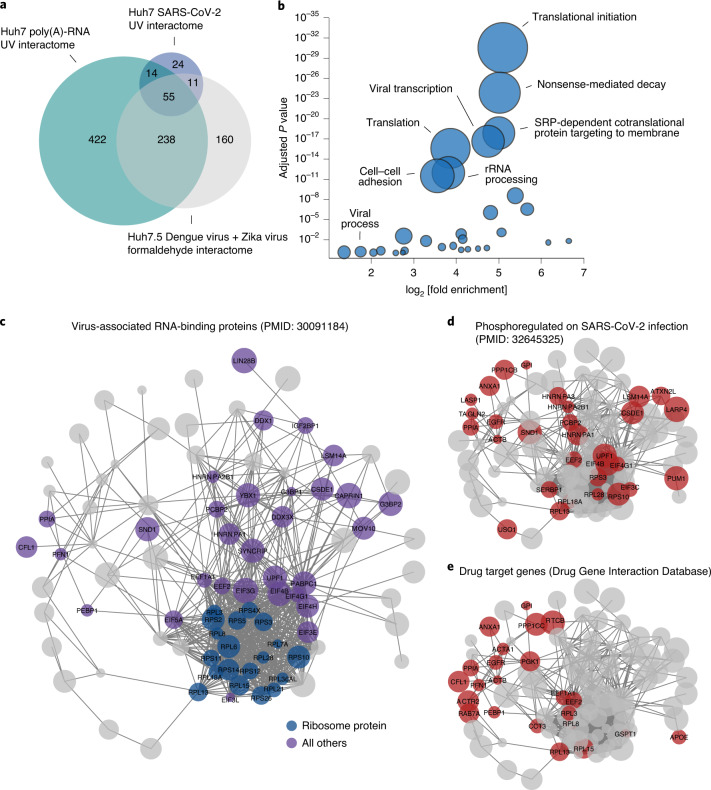
Viral RNA contacts regulators of RNA metabolism and host response. **a**, Intersection of the expanded SARS-CoV-2 RNA interactome with the poly(A)-RNA interactome and the Dengue/Zika virus interactome in Huh7 cells (Supplementary Table 2). **b**, GO enrichment analysis of SARS-CoV-2 RNA interactome proteins. Circle sizes scale to the number of detected proteins. SRP, signal recognition particle. Statistical test: Fisher’s exact test with Benjamini–Hochberg adjustment. **c**, Protein–protein association network of the expanded SARS-CoV-2 RNA interactome. Published virus-associated proteins are highlighted. Proteins without connections are not shown. **d**, As in **c** but proteins undergoing dynamic phosphorylation upon SARS-CoV-2 infection^7^ are highlighted. **e**, As in **c** but proteins that overlap known drug target genes (Drug Gene Interaction Database) are highlighted.

The expanded SARS-CoV-2 RNA interactome encompassed 104 human proteins and included 13 SARS-CoV-2-encoded proteins. The vast majority of the human RNA interactome proteins (100 proteins, 96%) have been identified previously in system-wide studies aimed at capturing proteins that crosslink to RNA^36^ (Supplementary Table 2). Comparing this expanded SARS-CoV-2 RNA interactome with the poly(A)-RNA interactome in Huh7 cells^37^, revealed high overlap between both datasets (69 proteins, 66%) (Fig. 2a). Next, we compared our direct SARS-CoV-2 RNA interactome with proteins that directly or indirectly associate with the RNA genomes of Dengue and Zika viruses in Huh7.5 cells^38^. Sixty-six proteins (63%) of the expanded SARS-CoV-2 RNA interactome also associated with the Dengue and Zika virus RNAs, while 38 proteins (36.5%) were unique SARS-CoV-2 RNA binders (Fig. 2a). Since coronaviruses form replication/transcription complexes (RTCs), we also compared the expanded SARS-CoV-2 RNA interactome to the protein content of murine coronavirus RTCs^39^ and found 64 shared proteins (Supplementary Table 2).

Finally, only 10 of the 332 human proteins that bound recombinant SARS-CoV-2 proteins in uninfected cells^10^ also bound directly to viral RNA in infected cells (Supplementary Table 2). These results highlight the importance of discriminating between protein–protein and RNA–protein interactions when dissecting the biology of SARS-CoV-2.

### Biological functions of SARS-CoV-2 RNA-binding proteins

To analyse the biological functions of SARS-CoV-2 RNA binders, we performed a hypergeometric gene ontology (GO) enrichment analysis on the expanded SARS-CoV-2 RNA interactome. We observed strong enrichment for GO terms linked to translational initiation (GO:0006413), nonsense-mediated decay (GO:0000184), signal-recognition particle-dependent cotranslational protein targeting to the membrane (GO:0006614) and viral transcription (GO:0019083) (Fig. 2b and Supplementary Table 3). Consistent with the enrichment of these GO terms, the importance of subgenomic mRNA translation at the endoplasmic reticulum membrane is well established for coronaviruses^40^. Further, nonsense-mediated mRNA decay was recently described as an antiviral mechanism targeting coronavirus RNAs^41^.

In agreement with the crucial role of mRNA translation, the expanded SARS-CoV-2 RNA interactome included 19 ribosomal proteins and 12 translation factors. Among the translation factors, the eukaryotic translation initiation factor 4F (EIF4F) components EIF4G1 and EIF4B are regulated by mammalian target of rapamycin (mTOR) signalling^42,43^. EIF4B is important for recruiting the 40S subunit to mRNA and both the phosphatidylinositol-3-kinase (PI3K)/mTOR and mitogen-activated protein kinase (MAPK) pathways target EIF4B to control its activity^43^. Inhibition of PI3K/Akt/mTOR signalling has been demonstrated to suppress SARS-CoV-2 replication in Caco2 cells^8^.

**Extended Data Fig. 2:**
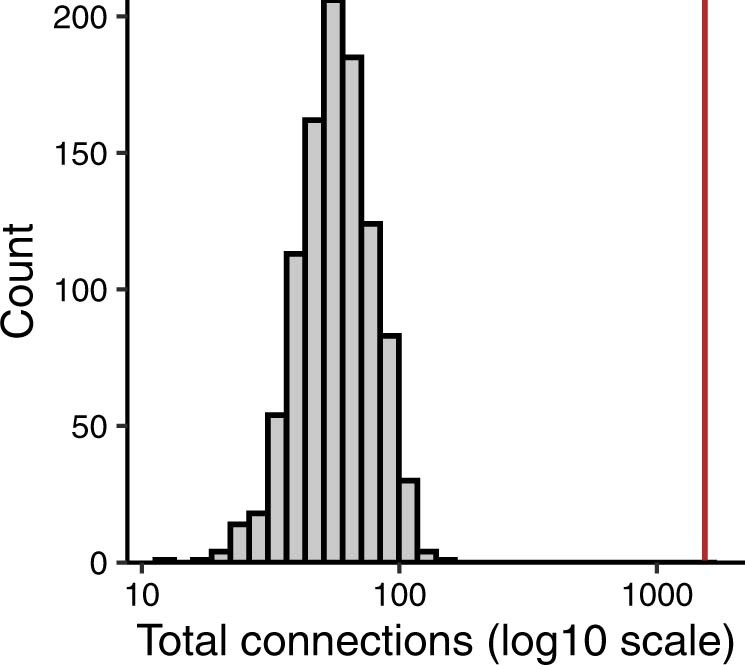
Connectivity in RAP-MS protein-protein association network. Total number of connections observed in protein-protein association network constructed based on expanded SARS-CoV-2 RNA interactome (red bar, 1,534 connections), compared to number of connections observed in random networks of equal size (grey bars, mean 60 connections, *z*-score 76) using random sampling of proteins detected in proteome measurements.

To examine the connectivity of the identified SARS-CoV-2 RNA-binding proteins and their relationship to virus-associated biological processes systematically, we constructed a protein–protein association network using our expanded RNA interactome (Fig. 2c and Supplementary Table 4). We observed a striking enrichment for physical interactions when comparing the total connectivity among RNA interactome proteins to the connectivity of equally sized networks sampled from expressed proteins (Extended Data Fig. 2 and Methods; permutation test *P* < 2.2 × 10^−16^). In addition to ribosomal proteins and translation factors, many virus-associated RNA-binding proteins are prominently represented in this network (Fig. 2c). Since RNA-binding proteins can be regulated by phosphorylation, we intersected our expanded SARS-CoV-2 RNA interactome with a recent phosphoproteomic dissection of SARS-CoV-2-infected cells^7^, highlighting 30 proteins that might be dynamically phosphorylated in response to SARS-CoV-2 infection (Fig. 2d).

We next integrated known drug–target interactions^44^ within this network and identified 23 SARS-CoV-2 RNA interactome proteins that can be targeted with existing compounds, including peptidyl-prolyl *cis*-*trans* isomerase A (PPIA), actin-related protein 2 (ACTR2; henceforth ARP2), sodium/potassium-transporting ATPase subunit alpha-1 (ATP1A1), annexin A1 (ANXA1), cofilin-1 (CFL1) and epidermal growth factor receptor (EGFR) (Fig. 2e). Notably, EGFR is a known target of compounds that inhibit SARS-CoV-2 replication^7,8,10^.

### Identification of activated host response pathways

**Fig. 3:**
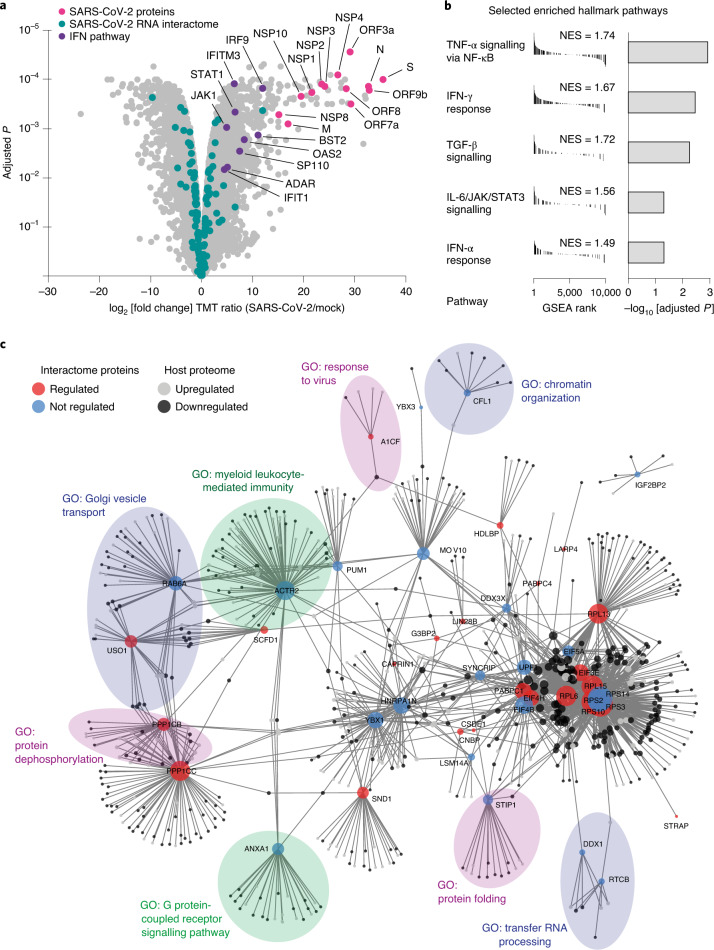
Connecting the SARS-CoV-2 RNA interactome to perturbations in host cells. **a**, Volcano plot of proteome abundance measurements in SARS-CoV-2-infected and uninfected Huh7 cells 24 h post-infection (*n* = 3) (Supplementary Table 5). Adjusted *P* value: two-tailed moderated *t*-test. SARS-CoV-2-encoded proteins are shown in magenta; human SARS-CoV-2 RNA interactome proteins are shown in teal; interferon response-related proteins are shown in purple. **b**, GSEA for the global proteome abundance measurements shown in **a**. Selected gene sets are shown; the full table displaying additional enriched gene sets is provided in Extended Data Fig. 3c. Statistical test: Kolmogorov–Smirnov test with Benjamini–Hochberg adjustment. NES, normalized enrichment score. **c**, Protein–protein association network of core SARS-CoV-2 RNA interactome proteins and their connections to differentially regulated proteins in SARS-CoV-2-infected cells based on curated interactions in STRING v.11 (ref. ^96^). Upregulated proteins are shown in light grey; downregulated proteins are shown in dark grey. Circle sizes scale to the number of connections of each interactome protein. Selected GO enrichments for network communities are shown in the transparent circles (Methods). Full GO term analysis is provided in Supplementary Table 8.

**Extended Data Fig. 3:**
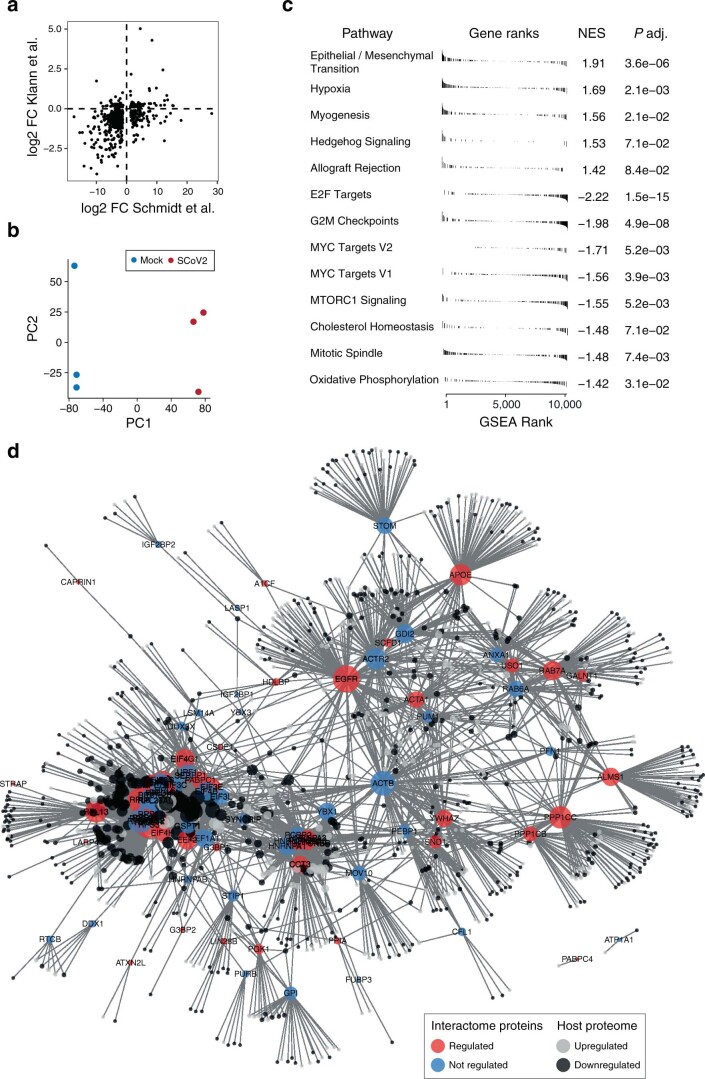
Proteome abundance changes in SARS-CoV-2 infected cells. **a**, Correlation of protein abundance measurements reported in Klann *et al*. and this study (r = 0.411). Proteins displayed are significant at an adjusted *P* value threshold of 0.01 in both studies (*n* = 712). **b**, Principle component analysis for proteome measurements of SARS-CoV-2 (SCoV2) infected or mock infected Huh7 cells. **c**, GSEA for proteins significantly regulated in global proteome measurements. Gene sets enriched in addition to those shown in Figure 3b are presented. Statistical test: Kolmogorov-Smirnov test with Benjamini-Hochberg adjustment. **d**, Protein-protein association network of expanded SARS-CoV-2 RNA interactome proteins (blue: interactome protein, not regulated; red: interactome protein, regulated) and their connections to differentially regulated proteins upon SARS-CoV-2 infection. Upregulated proteins are shown in light grey; downregulated proteins are shown in dark grey. Circle sizes scale to the number of connections of each interactome protein.

To gain deeper insight into host response pathways activated upon SARS-CoV-2 infection, we globally measured protein abundance changes in infected cells. We performed triplicate MS experiments on SARS-CoV-2-infected and uninfected Huh7 cells and identified 10,956 proteins with 2 or more unique peptides (Fig. 3a and Supplementary Table 5). Among the detected proteins, 4,578 proteins were regulated (adjusted *P* < 0.05, two-tailed *t*-test) after 24 h of SARS-CoV-2 infection, which is consistent with widespread proteome regulation and agrees well with previously published data (Extended Data Fig. 3a)^8,9^. As expected, proteome samples clustered according to their infection status in a principal component analysis (Extended Data Fig. 3b). Among differentially expressed proteins, we detected 13 viral proteins and 56 proteins from our expanded SARS-CoV-2 RNA interactome (Fig. 3a).

We next performed gene set enrichment analysis (GSEA) using our proteome abundance measurements. Among the most significantly enriched hallmark gene sets were ‘TGF-β signalling’, ‘TNF-α signalling via NF-κB’, ‘interferon (IFN)-γ response’ and ‘IL-6 JAK STAT3 signalling’ (Fig. 3b and Extended Data Fig. 3c), which is consistent with the induction of broad pro-inflammatory and antiviral responses in infected cells. Further, we observed significant enrichment of the gene sets ‘GO regulation of MAPK cascade’, ‘GO positive regulation of MAPK activity’ and ‘GO response to type I interferon’ (Supplementary Table 6). Recent evidence indicates that these pathways are indeed highly relevant in the context of SARS-CoV-2 infections^5,7,8^. Inhibition of growth factor signalling through the MAPK pathway, which responds to and controls the production of pro-inflammatory cytokines, including TNFα and IL-6, was shown to modulate SARS-CoV-2 replication^7,8^.

In agreement with recent transcriptome studies^5,6^, our proteome data suggest activation of interferon signalling upon SARS-CoV-2 infection. Among interferon-related genes, we observed significant upregulation of several major components of IFN signalling pathways, including *STAT1* and *IRF9*, which together with *STAT2* make up the interferon stimulated gene factor 3 (ISGF3) complex, their upstream components *TYK2* and *JAK1*, as well as their downstream targets *IFIT1*, *IFIT3*, *IFITM3*, *OAS2* and *ISG15* (Fig. 3a). Other strongly upregulated IFN-related genes include *BST2*, *SP110*, *UBE2L6*, *ADAR* and *TGIF1* (Supplementary Table 5). Notably, many SARS-CoV-2 RNA interactome members are linked to the IFN response. These include the strongly enriched PUM1 (ref. ^45^), YBX1 (ref. ^46^), SYNCRIP^47^, G3BP1 (refs. ^48,49^), G3BP2 (refs. ^48,49^), EIF4B^50^, MOV10 (ref. ^51^), CAPRIN1 (ref. ^49^), DDX3X^52^, LSM14A^53^, RyDEN^54,55^, STRAP^56^, ANXA1 (ref. ^57^), DDX1 (ref. ^58^), PCBP2 (ref. ^59^), HNRNPA2B1 (ref. ^60^) and YWHAZ^61^. In conclusion, our proteome analysis verifies the induction of an appropriate host response in SARS-CoV-2-infected Huh7 cells and further supports an important role for IFN and MAPK signalling in SARS-CoV-2 infection.

### Interplay between SARS-CoV-2 RNA binders and host cell proteins

As an RNA-based obligate intracellular parasite, SARS-CoV-2 must effectively interface with the host cell and rewire RNA metabolism and RNA-associated regulatory processes. In addition to controlling the RNA life cycle^62^, host RNA-binding proteins are an integral part of regulatory circuits that participate in host defence mechanisms^63,64^. To examine the interplay and connectivity between direct SARS-CoV-2 RNA binders and the host cell proteome, we used curated protein–protein interaction data to build a network that visualizes interactions between SARS-CoV-2 RNA binders and regulated host proteins (Fig. 3c, Extended Data Fig. 3d and Supplementary Table 7). We considered the connectivity among all differentially expressed host proteins and those that were detected in our core RNA interactome. Interactome proteins had a greater than twofold enrichment for network connections (mean 108) when compared to proteins not detected by RAP–MS (mean 45), indicating a significant enrichment in connectivity (Wilcoxon test, *P* = 8.92 × 10^−08^). To further contextualize this network, we overlaid biological processes that were enriched among regulated protein communities that associate with SARS-CoV-2 RNA binders (Fig. 3c and Supplementary Table 8). This analysis highlighted several cellular pathways and processes emerging as highly relevant in the context of SARS-CoV-2 infections, including myeloid-mediated immunity^65^, receptor signalling^8^, protein phosphorylation^7,8^, vesicle transport^8,10^, protein folding^6,7^ and translational regulation^8,66^.

Taken together, our network analysis connects RNA interactome proteins to emerging SARS-CoV-2 biology and provides a map of putative regulatory hubs in SARS-CoV-2 infections.

### Genetic screens identify functional SARS-CoV-2 RNA binders

**Fig. 4:**
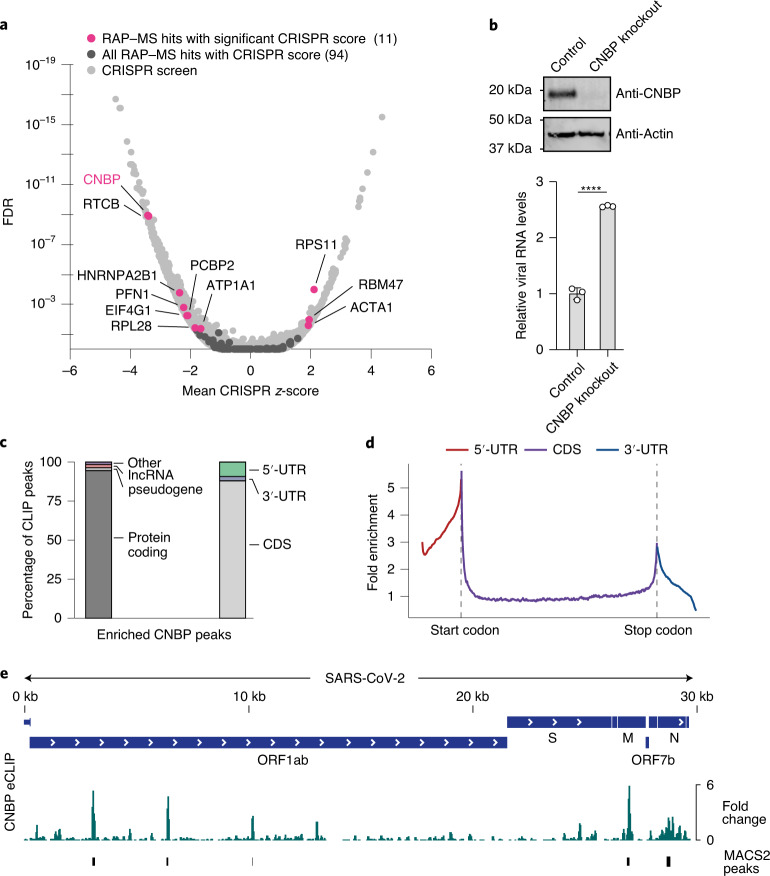
CNBP contacts SARS-CoV-2 viral RNA. **a**, SARS-CoV-2 RNA interactome proteins overlaid on genome-wide CRISPR perturbation data from SARS-CoV-2-infected Vero E6 cells^67^. Members of the expanded RNA interactome with significant (adjusted *P* < 0.05, two-sided *z*-test with Benjamini–Hochberg correction) changes in CRISPR *z*-scores are highlighted in magenta. The *y* axis is capped at 1 × 10^−19^, excluding 4 genes. **b**, Western blot of Huh7 CNBP knockout and control cell lines (top). RT–qPCR measurements of intracellular SARS-CoV-2 RNA (*RdRP* gene) at 48 h post-infection in Huh7 CNBP knockout and control cells (bottom). Quantification relative to 18S rRNA and control cells is shown. Values are the mean ± s.d. (*n* = 3 independent infections). *P* values were determined using an unpaired two-tailed *t*-test. ****P < 0.0001. **c**, Distribution of CNBP eCLIP peaks to different RNA types and transcript regions. **d**, Meta-gene analysis of CNBP eCLIP signal across mature mRNAs. **e**, CNBP eCLIP data aligned to the SARS-CoV-2 RNA genome. The fold change relative to the size-matched input is shown. MACS2-enriched peaks are shown below the fold change track. Source data

To functionally stratify our direct RNA binders, we intersected the SARS-CoV-2 RNA interactome with a recent genome-wide CRISPR perturbation screen designed to identify host factors that affect cell survival after SARS-CoV-2 infection^67^. Out of 104 human proteins in our expanded RNA interactome, we obtained CRISPR *z*-scores for 94 proteins^67^; depletion of 11 of these proteins had a statistically significant effect on SARS-CoV-2-induced cell death (Fig. 4a). Strikingly, cellular nucleic acid-binding protein (CNBP), the human protein most significantly enriched in RAP–MS, also had the most significant effect on virus-induced cell death among all SARS-CoV-2 RNA interactome members (Fig. 4a). In addition to the 11 aforementioned proteins, the direct SARS-CoV-2 RNA binders cold shock domain-containing protein E1 (CSDE1)^68^, polyadenylate-binding protein 1 (PABPC1) (refs. ^11,68^) and Ras-related protein Rab-7a (RAB7A)^11^ were also identified as host factors with functional relevance in SARS-CoV-2 infections by genetic screening approaches.

### CNBP functions as an antiviral regulator

CNBP is required to activate the innate immune response and has been linked to regulating the expression of pro-inflammatory cytokines in response to foreign nucleic acid sensing^69,70^. Notably, CNBP-deficient animals were highly susceptible to infections with different pathogens^69,70^. These findings are consistent with CNBP-depleted cells being sensitized to virus-induced cell death, which suggests that CNBP may act as an antiviral regulator. To corroborate the functional importance of CNBP in SARS-CoV-2 infections, we generated polyclonal Huh7 CNBP knockout cell lines using CRISPR–Cas9 (Fig. 4b). We infected CNBP knockout cells with SARS-CoV-2 and noted significantly elevated levels of intracellular viral RNA compared to matched Huh7 control cells (Fig. 4b). Thus, CNBP is indeed a functionally important SARS-CoV-2 RNA interactor.

To confirm the direct physical engagement of SARS-CoV-2 RNAs by CNBP, we performed enhanced crosslinking and immunoprecipitation (eCLIP) in SARS-CoV-2-infected Huh7 cells and quantified the enrichment of CNBP peaks relative to size-matched input libraries^71^. First, we analysed CNBP binding to the human transcriptome. Consistent with earlier reports^72^, CNBP bound to protein-coding transcripts and displayed a preference for binding within the coding sequence (CDS) of mRNAs (Fig. 4c,d). A large number of transcripts bound by CNBP in SARS-CoV-2-infected cells were previously reported as CNBP targets (approximately 46%; Supplementary Table 9). We next analysed CNBP binding to SARS-CoV-2 RNA and observed several strongly enriched binding sites in the viral genome (Fig. 4e). These data provide strong evidence for a direct interaction between CNBP and SARS-CoV-2 RNAs in infected cells and validate that RAP–MS indeed identifies direct RNA binders. Further, the finding that CNBP preferentially associates with the CDS of mature mRNAs lends credibility to its previously proposed role as a translational regulator^72^ in addition to its function in regulating pro-inflammatory cytokines.

### LARP1 binds genomic and subgenomic SARS-CoV-2 RNAs

Other than CNBP, two members of the La-related protein (LARP) family, namely LARP1 and LARP4, were strongly enriched in SARS-CoV-2 RNA purifications. While LARP1 did not quite meet our significance cut-off, both LARP1 and LARP4 were among the 15 host proteins with the strongest enrichment based on overall effect size, indicating that LARP1 is very likely a SARS-CoV-2 RNA binder. Additionally, LARP1 was detected among protein–protein interactors of the nucleocapsid protein in uninfected cells^10^.

**Fig. 5:**
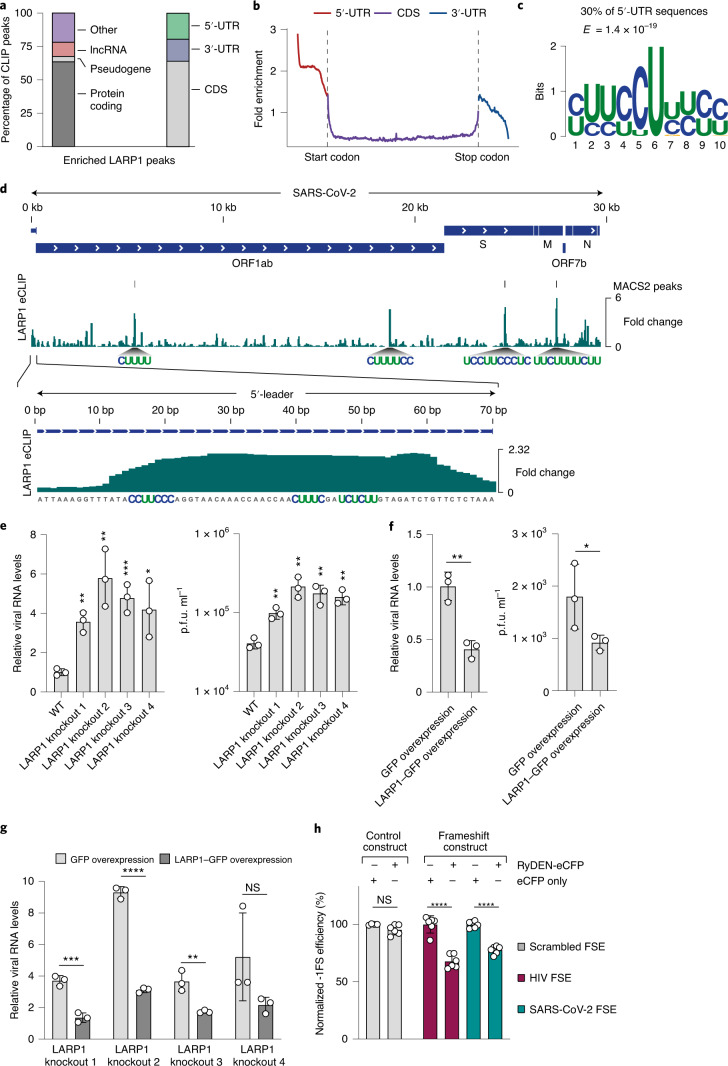
LARP1 binds SARS-CoV-2 RNAs and restricts viral replication. **a**, Distribution of LARP1 eCLIP peaks to different RNA types and transcript regions. **b**, Meta-gene analysis of LARP1 eCLIP signal across mature mRNAs. **c**, Oligopyrimidine-rich sequence motif discovered de novo in LARP1 peaks mapping to 5′-UTRs (Methods). **d**, LARP1 eCLIP data aligned to the SARS-CoV-2 RNA genome. The fold change relative to the size-matched input is shown. MACS2-enriched peaks are shown above the fold change track. Oligopyrimidine-rich sequences that coincide with strongly enriched LARP1 peaks are highlighted. A zoom-in to the SARS-CoV-2 5′-leader sequence is shown below the genomic alignment. **e**, Left: RT–qPCR measurements of intracellular SARS-CoV-2 RNA at 24 h post-infection in WT HEK293 cells or 4 different LARP1 knockout cell lines. Quantification relative to 18S rRNA and WT cells is shown. Right: Infectious viral titres in the supernatants of infected cells quantified by plaque assays at 24 h post-infection. *P* values were determined using an unpaired two-tailed *t*-test. **f**, Left: RT–qPCR measurements of intracellular SARS-CoV-2 RNA at 24 h post-infection in HEK293 cells transiently overexpressing GFP or LARP1–GFP proteins. Quantification relative to 18S rRNA and GFP-overexpressing cells is shown. Right: Infectious viral titres in the supernatants of infected cells quantified by plaque assays at 24 h post-infection. *P* values were determined using an unpaired one-tailed *t*-test. **g**, RT–qPCR measurements of intracellular SARS-CoV-2 RNA at 24 h post-infection in LARP1 knockout cells complemented with either GFP or LARP1–GFP plasmids. Quantification relative to 18S rRNA and GFP-transfected WT cells is shown. *P* values were determined using an unpaired two-tailed *t*-test. **e**–**g**, All values are the mean ± s.d. (*n* = 3 independent infections) **h**, Quantification of ribosomal frameshifting efficiency using a dual-fluorescence translation reporter (Extended Data Fig. 4d) in HEK293 cells is shown. Data were normalized to cells transfected with eCFP (*n* = 6 independent transfections, except for control RNA *n* = 4). Values are the mean ± s.d. ****P < 0.0001, ***P < 0.001, **P < 0.01, *P < 0.05; NS, not significant; FSE, frameshift element.

Given that LARP1 is a major downstream target of mammalian target of rapamycin complex 1 (mTORC1) (refs. ^73,74^) and inhibition of PI3K/Akt/mTOR was recently shown to inhibit SARS-CoV-2 replication in Caco2 cells^8^, we sought to characterize the LARP1-SARS-CoV-2 axis in greater detail. We performed eCLIP^71^ to map direct physical interactions between LARP1 and its RNA targets. LARP1 predominantly bound protein-coding transcripts and we observed most of the enriched peaks in the CDS, followed by 5′-UTR and 3′-UTR sequences (Fig. 5a). Previous work suggested that LARP1 binds the 7-methylguanosine triphosphate (m^7^Gppp) moiety of the cap and the adjacent 5′-terminal oligopyrimidine (5′TOP) motif of mRNAs to regulate their translation^75^. Consistent with this finding, our eCLIP data revealed a strong enrichment of 5′-proximal nucleotides in 5′-UTR sequences and we recovered an oligopyrimidine motif reminiscent of TOP-like sequences in approximately 30% of all bound 5′-UTRs (Fig. 5b,c). Out of 112 mRNAs that are regulated by LARP1 downstream of mTOR^76^, we observed LARP1 binding to 84 mRNAs (75%; Supplementary Table 10). In line with the known regulatory functions of LARP1 (ref. ^76^), LARP1 target transcripts were most strongly enriched for GO terms linked to translational regulation (Supplementary Table 11). Together, these data demonstrate that our eCLIP experiments recovered known regulatory interactions of LARP1.

Having confirmed the quality of our eCLIP experiment on host RNAs, we next characterized LARP1 binding to SARS-CoV-2 RNAs and found several regions of enrichment that coincided with oligopyrimidine sequences (Fig. 5d). Notably, we observed LARP1 binding to the first 70 nucleotides at the 5′-end of the SARS-CoV-2 genome, which corresponds to the viral 5′-leader sequence^77^ and contains a TOP-like motif instance (Fig. 5d). Binding to the 5′-leader, which is present in all viral subgenomic mRNAs, suggests a direct association of LARP1 with subgenomic mRNAs.

### LARP1 represses SARS-CoV-2 replication

**Extended Data Fig. 4:**
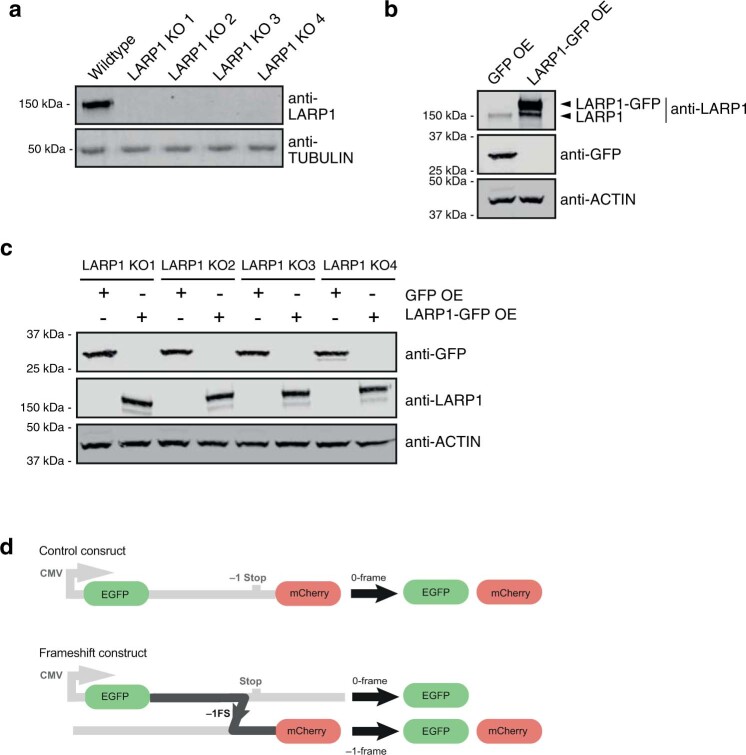
Functional validation of SARS-CoV-2 RNA binders. **a**, Western blot of WT HEK293 cells and four different HEK293 LARP1 knockout (KO) cell lines generated with CRISPR-Cas9 (see Methods). Expression of LARP1 was evaluated relative to Tubulin. **b**, Western blot of HEK293 cells transiently overexpressing (OE) GFP or LARP1-GFP proteins at 48 h post transfection. Arrows indicate endogenous LARP1 proteins and GFP-tagged LARP1. **c**, Western blot of four different HEK293 LARP1 knockout cell lines transiently transfected with plasmids encoding GFP or LARP1-GFP proteins at 48 h post transfection. Experiments were repeated at least two times. **d**, Schematic of dual-fluorescence translation reporter to quantify ribosomal frameshifting efficiency. The depicted control construct contains enhanced GFP (eGFP) and mCherry in an in-frame orientation, leading to the production of both fluorescent proteins separated by a self-cleaving 2A peptide when the 0 reading frame is translated. In the frameshift construct depicted below, eGFP and mCherry are separated by an in-frame stop codon, preventing the production of mCherry when the 0 reading frame is translated. −1FS leads to the production of eGFP and mCherry and the ratio between both fluorescent proteins is a direct measure of frameshifting efficacy. −1FS: –1 ribosomal frameshifting. Source data

To determine the impact of LARP1 depletion on SARS-CoV-2 replication, we generated four clonal LARP1 knockout cell lines using CRISPR–Cas9 in HEK293 cells (Extended Data Fig. 4a). We infected cells with SARS-CoV-2 and measured intracellular viral RNA levels and the production of infectious virus. Compared to wild-type (WT) cells, LARP1 knockout cells displayed approximately fivefold higher levels of intracellular viral RNA and a similar increase in the production of infectious virus (Fig. 5e). Conversely, transient overexpression of LARP1 fused to green fluorescent protein (GFP) in WT cells led to a significant reduction of viral RNA and infectious virus when compared to GFP expression alone (Fig. 5f and Extended Data Fig. 4b). Next, we complemented LARP1 knockout cells with transiently expressed LARP1–GFP proteins (Fig. 5g, Extended Data Fig. 4c). In all knockout cell lines, we observed a clear reduction in intracellular viral RNA that approached WT levels when compared to cells transfected with GFP alone. These experiments established that LARP1 functions as a repressor of SARS-CoV-2 replication in infected human cells.

### RyDEN suppresses ribosomal frameshifting during SARS-CoV-2 RNA translation

LARP1 interacts with PABPC1 and both LARP1 and PABPC1 have been proposed to reside in the same ribonucleoprotein complex with RyDEN^54^, all of which were enriched in RAP–MS experiments. In addition to being an IFN-induced protein, RyDEN suppresses Dengue virus production in infected cells^54^ and inhibits programmed -1 ribosomal frameshifting (-1FS) in human immunodeficiency virus type 1 (HIV-1) infections^55^.

In coronaviruses, production of RdRP requires translation of the *ORF1b* gene, which is controlled by -1FS. For SARS-CoV-2, it is presently unknown if the efficiency of -1FS is important for the viral life cycle^78^. To dissect if RyDEN can modulate the frequency of -1FS in SARS-CoV-2, we generated a dual-colour fluorescence reporter system to quantify frameshifting efficiency in response to RyDEN induction, as seen upon SARS-CoV-2 infection (Extended Data Fig. 4d and Methods). Using a reporter containing the HIV-1 frameshift element as a positive control, we confirmed that overexpression of RyDEN fused to enhanced cyan fluorescent protein (eCFP) suppressed -1FS when compared to eCFP expression alone (Fig. 5h). Importantly, overexpression of RyDEN also led to a significant reduction of -1FS during translation of the SARS-CoV-2 frameshift element (Fig. 5h). Together, our results show that RyDEN is induced upon SARS-CoV-2 infection, associates with the SARS-CoV-2 RNA in infected cells and modulates the efficiency of SARS-CoV-2 -1FS.

### Pharmacological inhibition of interactome proteins restricts viral replication

Next, we tested if targeting the SARS-CoV-2 RNA interactome and its associated pathways with known inhibitors is effective in restricting viral replication. We selected four inhibitors that target components of our expanded RNA interactome: PPIA; ARP2; ATP1A1; and DDX3X. While DDX3X is a DEAD-box RNA helicase and canonical RNA-binding protein, PPIA, ARP2 and ATP1A1 are non-classical RNA binders that are nonetheless robustly detected among RNA-binding proteins in Huh7 cells^36,37,79^. In addition to Huh7 cells, we evaluated all inhibitors in Calu3 cells, a human lung epithelial cell line that is naturally susceptible to SARS-CoV-2 infection.

**Fig. 6:**
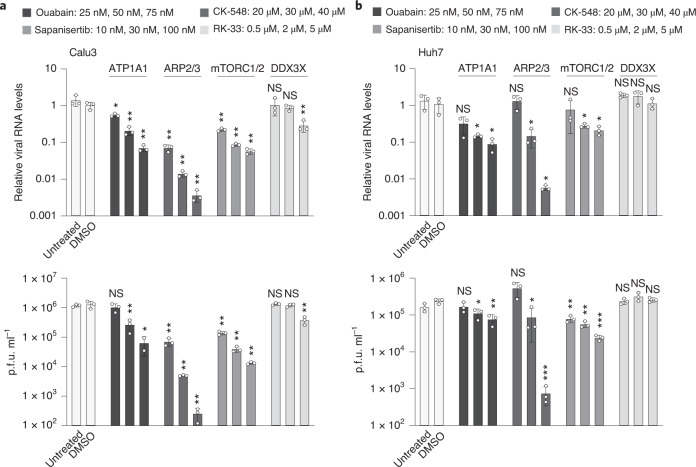
RNA interactome inhibitors reduce virus replication. **a**, Top: RT–qPCR measurements of intracellular SARS-CoV-2 RNA at 24 h post-infection in Calu3 cells after inhibitor treatment. Inhibitors were used at the indicated concentrations (left to right). Values were normalized to 18S rRNA measurements and compared to DMSO-treated cells. Bottom: Infectious viral titres in the supernatants of infected Calu3 cells quantified by plaque assays at 24 h post-infection. **b**, As in **a** but for Huh7 cells and at 48 h post-infection. All values are the mean ± s.d. (*n* = 3 independent infections). *P* values were determined using an unpaired two-tailed *t*-test. ***P < 0.001, **P < 0.01, *P < 0.05.

**Extended Data Fig. 5:**
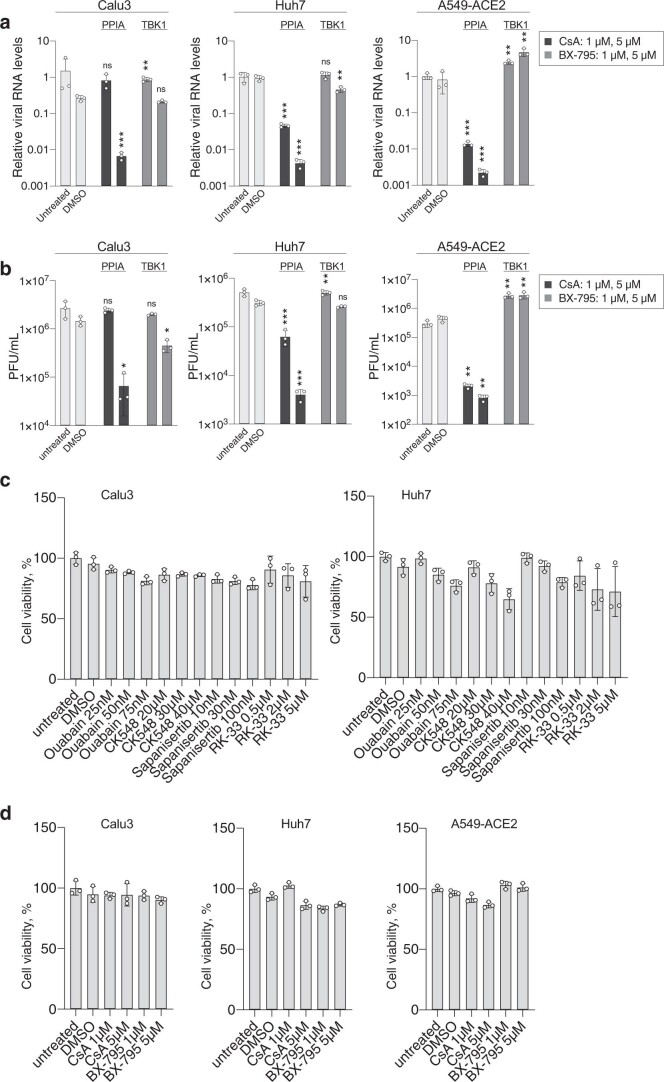
Pharmacological inhibition of SARS-CoV-2 RNA interactome proteins. **a**, RT-qPCR measurements of intracellular SARS-CoV-2 RNA (*RdRP* gene) in infected Calu3, Huh7 and A549-ACE2 cells after inhibitor treatment. Inhibitors were used at indicated concentration (left to right). Calu3 cells were assayed 24 h post-infection, Huh7 and A549-ACE2 cells were assayed 48 h post-infection. Values are normalized to 18S rRNA measurements and compared to untreated or DMSO treated cells. **b**, Infectious viral titers in the supernatants of infected Calu3, Huh7 and A549-ACE2 cells after inhibitor treatment. Inhibitors were used at indicated concentration (left to right). Calu3 cells were assayed 24 h post-infection, Huh7 and A549-ACE2 cells were assayed 48 h post-infection. All values in **a-b** are mean ± s.d. (*n* = 3 independent infections) **c-d**, Cell viability assay in inhibitor-treated and untreated cells. Values are the mean ± s.d. (*n* = 3 independent treatments). *P* values determined in unpaired two-tailed *t*-test. ***P < 0.001; **P < 0.01; *P < 0.05; ns, not significant.

We observed a dose-dependent inhibition of intracellular viral RNA expression accompanied by a reduction in the production of infectious virus for the PPIA inhibitor cyclosporin A (Extended Data Fig. 5a,b), the ARP2/3 complex inhibitor CK-548 and the ATP1A1 inhibitor ouabain (Fig. 6a,b). The observed effect was highly consistent between Calu3 and Huh7 cells (Fig. 6a,b). While CK-548 treatment reduced cell viability at the highest concentration in Huh7 cells, we did not observe such effects at identical concentrations in Calu3 cells. All other efficacious inhibitors had no apparent effect on cell viability (Extended Data Fig. 5c,d). Unlike the three aforementioned compounds, inhibition of DDX3X only led to a moderate reduction of intracellular viral RNA and infectious virus in Calu3 cells at the highest concentration (Fig. 6a,b).

Beyond inhibiting direct RNA binders, we also targeted mTORC1, the upstream regulatory complex that controls LARP1 activity. Consistent with LARP1 restricting SARS-CoV-2 replication, we observed that inhibiting mTORC1/2 resulted in reduced viral replication in Huh7 and Calu3 cells (Fig. 6a,b). These findings agree well with previous results showing that mTORC1 phosphorylates LARP1, which leads to a translational de-repression of LARP1 target mRNAs^76^. Indeed, recent phosphoproteomic surveys demonstrate that LARP1 undergoes dynamic phosphorylation in response to SARS-CoV-2 infection^7,8^.

Inhibition of another upstream regulator, TANK-binding kinase 1, which interacts with the SARS-CoV-2 RNA binders DDX3X^52^ and ANXA1 (ref. ^57^), increased the levels of viral RNA and infectious virus in A549-ACE2 cells, but did not show a consistent effect in Huh7 or Calu3 cells (Extended Data Fig. 5a,b). Together, our experiments demonstrate that RNA interactome proteins represent viable targets for inhibiting SARS-CoV-2 replication. The SARS-CoV-2 RNA interactome provides valuable starting points for future mechanistic studies and may help developing new antiviral approaches for COVID-19.

## Discussion

Decoding how the RNA genomes of pathogenic RNA viruses interface with the host cell proteome has been a long-standing challenge. In this study, we provide detailed molecular insights into the identity of host factors and cellular machinery that directly and specifically bind SARS-CoV-2 RNAs during infection of human cells. We integrate CRISPR perturbation data and perform genetic and pharmacological validation experiments that together suggest functional roles for 18 RNA interactome proteins in SARS-CoV-2 infections.

Beyond identifying proteins that bind SARS-CoV-2 RNAs, we globally mapped where CNBP and LARP1 contact viral and human RNA and report binding preferences that are consistent with previously described regulatory functions of both proteins. While we show that CNBP acts as an antiviral factor, it remains to be determined if its role as a regulator of mRNA translation or its effect on cytokine expression is critical for this function. We provide strong genetic evidence for a functional role of LARP1 in restricting SARS-CoV-2 replication. Remarkably, the SARS-CoV-2 5′-leader contains a TOP-like sequence motif that is bound by LARP1 in infected cells. While the TOP-like sequence is still several nucleotides away from the 5′-end of the SARS-CoV-2 leader, it is tempting to speculate that binding of LARP1 would negatively influence translation of SARS-CoV-2 RNAs similar to LARP1-mediated translational repression of host-encoded 5′TOP mRNAs.

In addition to genetic perturbation, we inhibited SARS-CoV-2 RNA binders pharmacologically. Notably, all host proteins and complexes that are effectively targeted by these inhibitors have previously been linked to viral diseases: (1) PPIA is involved in protein folding and has a well-documented impact on the replication of viruses^80,81^. Its direct interaction with SARS-CoV-2 RNA expands these previously described functions. While the PPIA inhibitor cyclosporin A has immunosuppressive properties, the non-immunosuppressive cyclosporin A analogue alisporivir may offer greater translational potential^82^; (2) a role for the RNA-binding metabolic enzyme ATP1A1^37^ in coronavirus and respiratory syncytial virus infections has been reported previously^83,84^. ATP1A1 had a significant effect on virus-induced cell death in a SARS-CoV-2 CRISPR perturbation screen^67^. Hence, both genetic and pharmacological evidence point to ATP1A1 as an important SARS-CoV-2 host factor; (3) ARP2 is part of the actin-related protein 2/3 complex and contributes to regulating cell shape and motility, which can affect intracellular pathogens^82^. ARP2 has been identified as a respiratory syncytial virus host factor and is involved in filopodia formation^85^. Recent work demonstrated that SARS-CoV-2 infection induced a dramatic increase in filopodia and viral particles localized to these actin-rich protrusions^7^.

In addition to the aforementioned factors, we observed various other notable proteins among SARS-CoV-2 RNA binders. These include vesicle trafficking proteins (SCFD1, USO1, RAB1A, RAB6D, RAB6A, RAB7A, GDI2), cytoskeleton regulators (ARP2, CFL1, PFN1, ACTA1), RNA editing cofactors (RBM47, A1CF) and subunits of a transfer RNA-splicing ligase complex (DDX1, RTCB).

Our work highlights opportunities for targeting proteins or pathways linked to the SARS-CoV-2 RNA interactome to interfere with viral infection. We believe that our approach provides a general roadmap for dissecting the biology of RNA viruses and the interactions between hosts and pathogens at the molecular level.

## Methods

### Tissue culture

We maintained Huh7, Calu3, HEK293, ACE2-A549 (a generous gift from A. Pichlmair) and TMPRSS2-Vero E6 cells (a generous gift from S. Pöhlmann) in DMEM medium (Thermo Fisher Scientific) supplemented with 10% heat-inactivated FCS (Thermo Fisher Scientific) and 100 U ml^−1^ streptomycin and 100 mg ml^−1^ penicillin. Cells were grown at 37 °C and 5% CO_2_.

### Generation of LARP1 knockout cell lines using CRISPR–Cas9

To generate the LARP1 CRISPR knockout cells, we used the pX335-U6-chimeric-BB-CBh-hSpCas9n(D10A) nickase (a generous gift from F. Zhang) together with GTTGGGTGGCAGTTTACGGGT and GCCACCCAAGAAGGACATGA as guide sequences. HEK293 cells were transfected with *Trans*IT-X2 (Mirus Bio) and selected with 2 µg ml^−1^ of puromycin in DMEM for 48 h and with 1 µg ml^−1^ for another 48 h. We picked single colonies and screened for LARP1 deletion by western blotting.

Plasmids for LARP1 overexpression were generated using C-terminal Myc-DDK-tagged human LARP1 (NM_015315), which was purchased from OriGene and subcloned into pEGFP-C1 retaining the C-terminal Myc-DDK tag.

### Generation of CNBP knockout cell lines using CRISPR–Cas9

A total of 2.5 × 10^5^ Huh7 cells per well were seeded in a 6-well plate and transfected the next day with 2.5 µg of a commercially available CNBP CRISPR–Cas9 knockout plasmid (catalogue no. sc-404090; Santa Cruz Biotechnology) using 2 µl of Lipofectamine 2000 (Thermo Fisher Scientific) per 1 µg of DNA. A plasmid containing a puromycin resistance gene was cotransfected as the selection marker. Control cells were transfected only with the puromycin resistance plasmid. Successfully transfected cells were selected with puromycin (5 µg ml^−1^) starting at 24 h post-transfection for 2 d. CNBP expression in polyclonal cell populations was analysed by western blot.

### Virus production

We used previously described patient-derived SARS-CoV-2 isolates^86,87^ propagated on Vero cells. To make high-titre viral stocks, we used TMPRSS2-Vero E6 cells, which were infected at a multiplicity of infection (MOI) of 0.005 plaque-forming units (p.f.u.) per cell for virus propagation. After 1 h of incubation at 37 °C, the inoculum was removed, cells were washed with PBS and OptiMEM (Gibco) containing 1% FCS was added. At 48 h post-infection, the cell culture supernatant was cleared by centrifugation (500*g* for 5 min) and aliquoted. Viral titres were determined by plaque assay on TMPRSS2-Vero E6 cells and by crystal violet staining.

### Virus infections

In general, the virus inoculum was prepared in DMEM containing 5% FCS and 100 U ml^−1^ of streptomycin and 100 mg ml^−1^ of penicillin. Cells were washed with PBS and incubated with the inoculum for 1 h at 37 °C. The inoculum was removed and fresh DMEM supplemented with 5% FCS; 100 U ml^−1^ of streptomycin and 100 mg ml^−1^ of penicillin were added to the cells.

### RAP–MS

RAP–MS was carried out as described previously^15^ with the following modifications: to capture endogenous SARS-CoV-2 RNAs, we designed and synthesized 5′ biotinylated 90-mer DNA oligonucleotides (Integrated DNA Technologies) antisense to the complementary target RNA sequence. We used 67 probes such that one probe binding site occurred roughly every 400 bases in the approximately 30-kilobase (kb) SARS-CoV-2 genome and excluded regions that matched to human transcripts or genomic regions as described previously^88,89^. For the SARS-CoV-2 RNA and RMRP antisense purifications, we grew ten 10-cm tissue culture plates of Huh7 cells per replicate. We prepared two replicates for SARS-CoV-2 RNA and RMRP purifications and included two non-crosslinked control samples that were used for RMRP purifications. SARS-CoV-2 infection was carried out with a previously described isolate^87^ at an MOI of 10 for 24 h. Cells were washed once with PBS and then crosslinked on ice using 0.8 J cm^−^^2^ of 254 nm UV light in a GS Gene Linker (Bio-Rad Laboratories). Cells were then lysed on the tissue culture plate by adding 1 ml of RAP lysis buffer (10 mM of Tris pH 7.5, 500 mM of LiCl, 0.5% dodecyl maltoside, 0.2% SDS, 0.1% sodium deoxycholate, EDTA-free protease inhibitor cocktail (Thermo Fisher Scientific) and murine RNase inhibitor (New England Biolabs)). Lysates were then collected and flash-frozen in liquid nitrogen for storage at −80 °C. All subsequent steps were carried out as described previously^15^.

### RAP–MS protein digestion and TMT labelling

RAP-captured proteins were resuspended in 40 μl of 8 M of urea in 50 mM of Tris-HCl, followed by reduction with 4 mM of dithiothreitol (DTT) for 30 min at room temperature and alkylation with 10 mM of iodoacetamide for 45 min at room temperature in the dark. All six samples were then digested with 0.1 μg of Lys-C for 2 h, followed by a reduction of the urea concentration to <2 M and continued digestion with 0.5 μg of trypsin overnight. Reactions were quenched with formic acid at a final concentration of 5% and then desalted by reverse-phase C18 stage tips as described previously^90^ and dried down. Peptides were then resuspended in 50 μl of 50 mM of HEPES buffer and isobarically labelled using 400 μg of TMT 6-plex (TMT6) isobaric labelling reagent (Thermo Fisher Scientific). The labelling reactions were then quenched with 4 μl of 5% hydroxylamine; samples were mixed together and dried. The sample was fractionated by SCX stage tip strategy using three pH cuts at 5.15, 8.25 and 10.3 as described previously^90^.

### Proteome analyses of SARS-CoV-2-infected cells

For the proteome measurements, we expanded the Huh7 cells to two 10-cm tissue culture plates per replicate. Cells were infected with a previously described SARS-CoV-2 isolate^87^ at an MOI of 10 and incubated for 24 h before being collected. Three process replicates of infected and non-infected cell line samples were generated. Cells were lysed in 8 M of urea, 75 mM of NaCl, 50 mM of Tris pH 8.0, 1 mM of EDTA, 2 µg ml^−1^ aprotinin, 10 μg ml^−1^ of leupeptin, 1 mM of phenylmethylsulfonyl fluoride, 10 mM of NaF, phosphatase inhibitor cocktail 2 (PIC2) (Sigma-Aldrich), PIC3 (Sigma-Aldrich) and 10 mM of sodium butyrate. Benzonase was added to digest nucleic acids and DNA was sheared using a probe sonicator (10% amplitude, 0.7 s on, 2.3 s off, 6 min 15 s total). Cell debris was removed by centrifugation and lysates were flash-frozen for storage. All samples were prepared for MS analysis using an optimized workflow as described previously^91^. Briefly, lysed samples were reduced, alkylated and digested by LysC for 2 h, followed by overnight digestion with trypsin. Digestions were quenched with formic acid and all peptide samples were desalted using reverse-phase C18 Sep-Pak cartridges. Samples were then quantified using the Pierce bicinchoninic acid protein assay and measured into 500-μg aliquots for isobaric labelling. Peptides were isobarically labelled with TMT6 following the reduced TMT protocol^92^. After confirming 98% or greater label incorporation, samples were mixed together and desalted. The resulting sample was then fractionated by offline high pH reversed-phase chromatography and concatenated into 24 fractions for analysis using online LC–MS/MS^91^.

### LC–MS/MS analysis (RAP–MS and proteome)

All the samples were analysed either on an Orbitrap Exploris 480 (RAP–MS fractions) or a Q Exactive Plus (proteome fractions) mass spectrometer coupled with an Easy nLC 1200 ultra-high pressure liquid chromatography system (Thermo Fisher Scientific) with solvent A of 0.1% formic acid/3% acetonitrile and solvent B of 0.1% formic acid/90% acetonitrile. One microgram of each of the proteome fractions and half of each of the RAP–MS fractions were injected on a 75-μm ID PicoFrit column packed in-house to approximately 28-cm length with ReproSil-Pur C18-AQ 1.9-μn beads (Dr. Maisch). Samples were separated at a 200 nl min^−1^ flow rate with a gradient of 2–6% solvent B for 1 min, 6–30% B for 84 min, 30–60% B for 9 min, 60–90% B for 1 min, followed by a hold at 90% B for 5 min. Both mass spectrometers were operated in data-dependent acquisition mode. An Exploris 480 MS1 scan (*r* = 50,000) was followed by MS2 scans (*r* = 15,000) for the top 20 most abundant ions using normalized automatic gain control (AGC) of 100% for MS1 and 200% for MS2, MS2 maximum injection time of 150 ms, normalized collision energy of 34 and fit filter of 50%. The Q Exative Plus MS parameters were set as follows: MS1, *r* = 70,000; MS2, *r* = 17,500; MS1 AGC target of 3e6; MS2 for the 12 most abundant ions using an AGC target of 5e4 and maximum injection time of 120 ms; and normalized collision energy of 29.

### Quantification and identification of peptides and proteins (RAP–MS and proteome)

MS/MS spectra were searched on the Spectrum Mill MS Proteomics Workbench against a Reference Sequence (RefSeq)-based sequence database containing 41,457 proteins mapped to the human reference genome (hg38) obtained via the University of California, Santa Cruz Table Browser (https://genome.ucsc.edu/cgi-bin/hgTables) on 29 June 2018, with the addition of 13 proteins encoded in the human mitochondrial genome, 264 common laboratory contaminant proteins, 553 human non-canonical small ORFs, 28 SARS-CoV-2 proteins obtained from RefSeq derived from the original Wuhan-Hu-1 China isolate (NC_045512.2) (ref. ^93^) and 23 new unannotated SARS-CoV-2 ORFs whose translation is supported by ribosome profiling^94^, yielding a total of 42,337 proteins. Among the 28 annotated SARS-CoV-2 proteins, we opted to omit the full-length ORF1ab to simplify peptide-to-protein assignment, and instead represented ORF1a and ORF1ab as the mature 16 individual NSPs that resulted from proteolytic processing of the 1a and 1ab polyprotein. Finally, we added to the database the UniProt entry for ORF9b. We further added the D614G variant of the SARS-CoV-2 spike protein that is commonly observed in European and American virus isolates. Spectrum Mill search parameters included: instrument setting of ESI-QEXACTIVE-HCD-v4-35-20; parent and fragment mass tolerance of 20 parts per million; trypsin allow P enzyme setting; and up to 4 missed cleavages. Carbamidomethylation and TMT labelling at lysine (with and without labelling at the N terminus) were set as fixed modifications, while variable modifications included acetylation of protein N termini, oxidized methionine, deamidation of asparagine and pyroglutamic acid at the peptide N-terminal glutamine. Peptide spectrum match score thresholding was optimized to achieve a target–decoy FDR of 1.2% for the validation of spectra. Peptide-level auto-validation was followed by protein polishing with an FDR of 0% at the protein level and a minimum score of 13.

The Spectrum Mill generated proteome-level export from the RAP–MS and proteome datasets, which were filtered for human proteins identified by two or more distinct peptides, SARS-CoV-2 proteins and unannotated virus ORFs, were used for further statistical analyses. Five of the SARS-CoV-2 NSPs (NSP6, NSP15, NSP16, NSP9 and NSP1) identified by a single, highly scoring distinct peptide were kept in the dataset. Keratins were excluded from the RAP–MS data. Protein quantification was achieved by taking the ratio of TMT reporter ion for each sample/channel over the median of all six channels. A moderated two-sample *t*-test was applied to compare SARS-CoV-2 RNA and RMRP samples after mean normalization and SARS-CoV-2-infected and non-infected samples after median-median absolute deviation (MAD) normalization of RAP–MS and proteome datasets, respectively. A Benjamini–Hochberg-corrected *P* value threshold of 0.05 was used to assess significantly regulated proteins in each of the datasets.

### Covalent protein capture and sequencing of crosslinked RNA

To capture RNA sequences covalently crosslinked to proteins purified with RAP–MS, we carried out RAP as described above. After our pilot RAP–MS experiment (Extended Data Fig. 1a,b), SARS-CoV-2-bound proteins were eluted from streptavidin beads by heat fragmentation of RNA (3 min at 91 °C in 100 mM of HEPES pH 7.5, 5 mM of MgCl_2_, 100 mM of KCl, 0.02% Triton X-100). For subsequent RAP–MS experiments, we replaced heat fragmentation with ribonuclease (RNase) H digestion, using 7.5 μl of RNase H (New England Biolabs), 2 μl of TURBO DNase (Thermo Fisher Scientific) in 55.5 μl of RNase H buffer (100 mM of HEPES pH 7.5, 75 mM of NaCl, 3 mM of MgCl_2_, 0.125% *N*-lauroylsarcosine (NLS), 0.025% sodium deoxycholate, 2.5 mM of tris(2-carboxyethyl)phosphine (TCEP)) and incubating for 30 min at 37 °C. Following the elution of proteins, supernatants were transferred into new tubes and beads were washed once with RNase H buffer. Wash fractions were pooled with eluates and stored on ice. The next steps were described previously in similar form by Quinodoz et al.^95^. We separated 100 μl of NHS magnetic beads (Thermo Fischer Scientific) on a magnet and discarded the supernatant. We then washed them with 1 ml of 1 mM of ice-cold HCl, followed by a quick rinse in 1 ml of ice-cold PBS. After removing the PBS, we immediately added the stored eluates to the prepared beads. Binding was carried out overnight at 4 °C on a rotating wheel. The next day, we quenched the NHS beads by adding 1 ml of 0.5 M Tris pH 8.0 and incubating for 1 h at 4 °C. We then washed the beads 4 times in 1 ml of modified RLT buffer (RLT buffer supplied by QIAGEN with added 10 mM of Tris pH 7.5, 1 mM of EDTA, 1 mM of EGTA, 0.2% NLS, 0.1% Triton X-100 and 0.1% NP-40). Next, we washed the beads twice more in 1 ml of 1× PBS, 5 mM of EDTA, 5 mM of EGTA, 5 mM of DTT, 0.2% Triton X-100, 0.2% NP-40, 0.2% sodium deoxycholate and incubated each washing step for 5 min at 55 °C. These heated washing steps were followed by two additional washes in the same buffer at room temperature. Subsequently, beads were rinsed on the magnet in 1× FastAP buffer (10 mM of Tris pH 7.5, 5 mM of MgCl_2_, 100 mM of KCl, 0.02% Triton X-100). Next, end repair was carried out by resuspending the beads in 50 μl of FastAP mix (39 μl of H_2_O, 5 μl of 10× FastAP buffer (Thermo Fisher Scientific), 1 μl of murine RNase inhibitor, 5 μl of FastAP enzyme (Thermo Fisher Scientific) and incubating for 20 min at 37 °C. In the meantime, we prepared 150 μl of T4 polynucleotide kinase (PNK) mix (120 μl of H_2_O, 20 μl of 10× T4 PNK buffer (New England Biolabs), 1 μl of murine RNase inhibitor, 7 μl of T4 PNK, 1 μl of TURBO DNase), which was added to the FastAP reaction and incubated for another 20 min at 37 °C. After end repair, we washed the beads once in modified RLT buffer and twice in detergent wash buffer (20 mM of Tris pH 7.5, 50 mM of NaCl, 0.2% Triton X-100, 0.2% NP-40, 0.2% sodium deoxycholate). We then rinsed the beads on the magnet twice with 1× T4 RNA ligase buffer (50 mM of Tris-HCl pH 7.5, 10 mM of MgCl_2_), before resuspending the beads in 25 μl of RNA ligation mix (9 μl of H_2_O, 3 μl of 10× T4 RNA ligase buffer (New England Biolabs), 0.3 μl of 0.1 M of ATP, 0.8 μl of dimethylsulfoxide (DMSO), 0.4 μl of murine RNase inhibitor, 9 μl of polyethylene glycol 8000 and 2.5 μl of T4 RNA ligase I High Concentration (New England Biolabs). Next, we added 5 μl of 20 nM of RiL19 (/5phos/rArGrArUrCrGrGrArArGrArGrCrGrUrCrGrUrG/3SpC3/; Integrated DNA Technologies) and incubated the samples for 75 min at 23 °C. After 3′-ligation, we washed the beads once in 1 ml of modified RLT buffer, followed by two washes in detergent wash buffer. Next, we resuspended the beads in 250 μl of proteinase K (New England Biolabs) mix containing 200 μl of NLS RNA elution buffer (20 mM of Tris pH 8.0, 10 mM of EDTA, 2% NLS, 2.5 mM of TCEP), 12.5 μl of 5 M of NaCl, 1 μl of 500 mM of TCEP, 12.5 μl of proteinase K and 24 μl of H_2_O and incubated the samples for 1.5 h at 55 °C. After proteinase K digestion, we separated beads on a magnet, transferred the supernatant into a new tube and extracted RNA using phenol-chloroform extraction. All subsequent manipulation steps were carried out as described in the eCLIP library preparation protocol^71^, starting with the reverse transcription of recovered RNA fragments.

### eCLIP

For the eCLIP experiments, we grew approximately 24 × 10^6^ Huh7 cells and infected them with SARS-CoV-2 at an MOI of 5 PFU per cell; 24 h after infection, culture medium was removed, cells were briefly rinsed with PBS and subjected to UV irradiation with a total dose of 0.8 J cm^−2^. Cells were scraped in PBS using a flexible rubber scraper, pelleted at 200*g* for 5 min and lysed by adding 2× CLIP lysis buffer (100 mM of Tris-HCl pH 7.4, 300 mM of NaCl, 2 mM of EDTA, 2% (v/v) NP40, 1% sodium deoxycholate, 0.5 mM of DTT). After a 30-min incubation at room temperature, lysates were stored at −80 °C.

Frozen lysates were combined with an equal amount of nuclease-free water to adjust the lysis buffer to a 1× concentration. Subsequent steps were performed as described in the eCLIP protocol^71^ with the following modifications. Immunoprecipitates were washed twice in 1 ml of CLIP lysis buffer, twice in immunoprecipitation wash buffer (50 mM of Tris-HCl pH 7.4, 300 mM of NaCl, 1 mM of EDTA, 1% (v/v) NP40, 0.5% sodium deoxycholate, 0.25 mM of DTT), followed by two washes in 50 mM of Tris-HCl pH 7.4, 1 mM of EDTA and 0.5% (v/v) NP40. All other steps were carried out as described in the eCLIP method^71^. We used the following antibodies for the immunoprecipitation reactions: CNBP antibody (catalogue no. 67109-1-Ig; Proteintech) and LARP1 (catalogue no. A302-087A; Bethyl Laboratories).

### Inhibitor treatment and infection

A total of 1 × 10^5^ Huh7 or A549-ACE2 cells or 3 × 10^5^ Calu3 cells were seeded per well of a 24-well plate. After 24 h (for Huh7 and A549-ACE2 cells) or 48 h (for Calu3 cells), the growth medium was replaced by DMEM with 5% FCS containing cyclosporin A (catalogue no. SML1018; Sigma-Aldrich), BX-795 hydrochloride (catalogue no. SML0694; Sigma-Aldrich), Ouabain octahydrate (catalogue no. O3125; Sigma-Aldrich), CK-548 (catalogue no. ALX-270-504-M002; Enzo Life Sciences), sapanisertib (catalogue no. HY-13328; Hölzel Diagnostika) or RK-33 (catalogue no. TMO-T6970; Hölzel Diagnostika) at the indicated concentrations (Fig. 6) 2 h before infection. Cells were infected with SARS-CoV-2 at an MOI of 0.5 PFU per cell (Huh7 and A549-ACE2 cells) or an MOI of 0.1 PFU per cell (Calu3 cells). After incubating the cells for 1 h with the virus inoculum, the medium was replaced with inhibitor-containing DMEM with 5% FCS. At the indicated time points post-infection, the supernatants were collected for plaque assay analyses and cells were lysed for quantitative PCR with reverse transcription (RT–qPCR) analyses.

### Infection of Huh7 CNBP knockout cells

A total of 1 × 10^5^ cells were seeded per well of a 24-well plate. The next day, cells were infected with SARS-CoV-2 at an MOI of 0.5 PFU per cell as described above. At the indicated time points post-infection, supernatants were collected for plaque assay analyses and cells were lysed for RT–qPCR analyses.

### Infection of HEK293 LARP1 knockout cells

A total of 1.5 × 10^5^cells were seeded per well of a poly-L-lysine-coated 24-well plate. For the rescue experiments, cells were transfected the next day with 500 ng per well LARP1-GFP overexpression plasmid or pEGFP-C1 as the control using 3 µl of *Trans*IT-X2 transfection reagent per 1 µg of DNA. At 24 h post-transfection, cells were infected with SARS-CoV-2 at an MOI of 0.5 PFU per cell as described above. At the indicated time points post-infection, supernatants were collected for the plaque assay analyses and cells were lysed for the RT–qPCR analyses.

### RNA extraction and RT–qPCR

Cells were lysed in 300 µl of TRIzol per well and RNA was extracted using the Direct-zol RNA Microprep Kit (Zymo Research). RNA was reverse-transcribed into complementary DNA using the AffinityScript Multiple Temperature Reverse Transcriptase system (Agilent Technologies) according to the manufacturer’s instructions. Viral RNA was quantified by qPCR using the PowerUp SYBR Green Master Mix (Thermo Fisher Scientific) and primers specific to the SARS-CoV-2 *RdRP* gene (forward: GTGARATGGTCATGTGTGGCGG, reverse: CARATGTTAAASACACTATTAGCATA) and 18S ribosomal RNA (forward: ATGGCCGTTCTTAGTTGGTG, reverse: GAACGCCACTTGTCCCTCTA). We calculated the differences in RNA expression using the $\Delta\Delta^{C_T}$ method versus 18S. To achieve power to detect small effects in gene expression, we performed four technical qPCR replicates from the same cDNA and took the median value for further analysis.

### Western blot

In general, we added NuPAGE LDS Sample Buffer (Thermo Fisher Scientific) to a 1× concentration and incubated samples for 3 min at 95 °C. Proteins were resolved by SDS–polyacrylamide gel electrophoresis using NuPAGE 4 to 12% Bis-Tris-HCl Gels (Thermo Fisher Scientific) at 200 V for 1 h, followed by transfer to a nitrocellulose membrane using the iBlot dry blotting system (Thermo Fisher Scientific). Western blots were performed using the iBind Automated Western System (Thermo Fisher Scientific). For protein detection, we used the following primary antibodies: nucleocapsid protein (catalogue no. ab272852; Abcam); POP1 (catalogue no. 12029-1-AP; Proteintech); LARP1; CNBP; α-Tubulin (catalogue no. 2144; Cell Signaling Technology); β-Actin (catalogue no. sc-47778; Santa Cruz Biotechnology). We used the following secondary antibodies: IRDye 800CW goat anti-rabbit IgG (LI-COR); IRDye 800CW goat anti-mouse IgG (LI-COR). For the visualization of bands, we used the Odyssey Clx Infrared Imager System (LI-COR).

### Plaque assay

TMPRSS2-Vero E6 cells were infected with 10-fold serial dilutions of the virus-containing sample in DMEM with 1% FCS. After a 1-h incubation, the inoculum was removed and cells were overlayed with 0.6% (w/v) methylcellulose (Carl Roth) in MEM (Gibco) supplemented with 25 mM of HEPES, 0.44% NaHCO_3_, 2 mM of GlutaMAX (Gibco), 100 U ml^−1^ of streptomycin, 100 mg ml^−1^ of penicillin and 5% FCS. At 4 d post-infection, cells were fixed and stained by adding 2× staining solution (0.23% crystal violet, 8% formaldehyde, 10% ethanol) directly to the medium for 2 h. Cells were washed with H_2_0 and plaques were counted to determine viral titres.

### Cell viability assay

For the cell viability assays, cells were seeded in 96-well plates (2 × 10^4^ cells per well for Huh7 and A549-ACE2 cells, 6 × 10^4^ cells per well for Calu3 cells) and treated with inhibitors as described for the infection assays. After 24 h (Calu3 cells) or 48 h (Huh7 and A549-ACE2 cells) of treatment, cell viability was assessed using the CellTiter-Glo reagent (Promega Corporation) according to the manufacturer’s instructions.

### Quantification of ribosomal frameshifting

HEK293 cells were transiently transfected with either the control or frameshifting construct of our dual-colour enhanced GFP (eGFP)–mCherry translation reporter outlined in Extended Data Fig. 4d. Briefly, cells transfected with this reporter express a single fluorescent protein (eGFP) when the 0 reading frame is translated (Extended Data Fig. 4d). Expression of a second fluorescent protein (mCherry) downstream of eGFP is dependent on -1FS, which prevents translation of an inframe stop codon. Thus, the ratio between mCherry and eGFP directly correlates to -1FS efficiency. As a normalization control, we used a construct lacking a stop codon in the 0 reading frame, leading to the expression of eGFP and mCherry in equal ratios.

RyDEN was expressed as fusion protein with eCFP. In the control experiments, a plasmid only carrying eCFP was used. Using flow cytometry (Novocyte Quanteon), eCFP^+^ cells were analysed for the ratio between mCherry and eGFP, providing a direct readout of ribosomal frameshifting efficiency. Accordingly, frameshifting efficiency was calculated using the ratio of mCherry to eGFP observed with the frameshifting reporter construct relative to the mCherry/eGFP ratio observed with the control construct (Extended Data Fig. 4d).

### Computational analyses

#### Protein–protein interaction network

To establish protein–protein interactions for the proteins identified from the MS experiments, we utilized STRING v.11 (ref. ^96^). For all network and interaction inferences, we used the ‘combined score’ from STRING, which utilizes both physical and functional interactions. Specifically, for the RAP–MS network (Fig. 2), we seeded all proteins detected with an adjusted *P* < 0.2 and positive log fold change from the moderated *t*-test between SARS-CoV-2 RNA and RMRP purifications. The edges between interacting proteins were included for those above a combined interaction score of 550. To generate the combined RAP–MS and proteome MS network, we seeded nodes where the adjusted *P* < 0.05 for either of the assays. Edges between RAP–MS and proteome MS nodes were included for combined interaction scores exceeding 700.

#### Gene set and pathway enrichment analysis

First, we performed a hypergeometric GO enrichment analysis for the expanded SARS-CoV-2 RNA interactome proteins using the DAVID tool (v.6.8, https://david.ncifcrf.gov/tools.jsp) and applying default settings (Fig. 2b). Additionally, we performed GSEA for the proteome experiments with the clusterProfiler R package (v.3.18)^97^ utilizing the Hallmark and C5 biological processes gene sets available through Molecular Signatures Database (v.7.2)^98^ (Fig. 3b). Genes were ranked based on the product of the log_2_ fold change and the log_10_ moderated *t*-test *P* value between SARS-CoV-2 and mock treatments. To establish enriched terms for communities within the interactome network (Fig. 3c), we considered all regulated genes in the proteome measurements interacting with a specific direct binder and computed enrichments using the C5 biological processes gene sets.

#### eCLIP and RNA sequencing analysis

Paired-end sequencing reads from (1) eCLIP experiments or (2) sequencing of crosslinked RNA fragments after RAP–MS, were trimmed using a custom Python script that simultaneously identified the unique molecular identifier associated with each read. These trimmed reads were then aligned to the SARS-CoV-2 reference genome (NC_045512.2 contig) using the Burrows–Wheeler Aligner (v.0.7.17)^99^. Next, we removed PCR duplicates using the unique molecular identifier-aware deduplication functionality in Picard’s MarkDuplicates (v.2.22.0). Finally, enriched regions of protein binding were identified using model-based analysis of ChIP–seq 2 (MACS2 v.2.2.7)^100^ to model the fold change between per-million fragment normalized counts (signal per million mapped reads) of the treated and control samples. Visualizations of the region were rendered from the PCR-deduplicated .bam files using the Integrative Genomics Viewer. CLIP peak annotations and overlaps were determined using custom functions and the GenomicRanges package (v.1.40.0)^101^. Meta-gene enrichment plots were computed using deepTools (v.3.4.3)^102^. To establish the binding motif of the LARP1 CLIP peaks, we performed a de novo motif enrichment using MEME (v.5.2).^103^ via a strand-aware sequence enrichment for peaks that overlapped a single 5′-UTR in our hg38 reference.

### Reporting Summary

Further information on research design is available in the Nature Research Reporting Summary linked to this article.

## Supplementary information

Reporting Summary

Supplementary Table 1 Proteins detected by quantitative mass spectrometry in SARS-CoV-2 RNA and RMRP RNA antisense purifications in infected human cells.

Supplementary Table 2 Intersection of the expanded SARS-CoV-2 RNA interactome with published work.

Supplementary Table 3 Gene Ontology enrichment analysis for the expanded SARS-CoV-2 RNA interactome. Statistical test: Fisher’s exact test with Benjamini–Hochberg adjustment.

Supplementary Table 4 Protein–protein association network based on STRING v.11 interactions between human proteins in the expanded SARS-CoV-2 RNA interactome.

Supplementary Table 5 Proteome abundance measurements in SARS-CoV-2 infected and uninfected Huh7 cells.

Supplementary Table 6 Gene set enrichment analysis for proteome abundance measurements in SARS-CoV-2 infected Huh7 cells (Hallmark and MSigDB C5 gene sets). Statistical test: Kolmogorov–Smirnov test with Benjamini–Hochberg adjustment.

Supplementary Table 7 Protein–protein association network based on STRING v.11 interactions between human proteins in the core SARS-CoV-2 RNA interactome and proteins regulated upon SARS-CoV-2 infection in Huh7 cells.

Supplementary Table 8 Gene Ontology enrichment analysis for protein–protein association network of core SARS-CoV-2 RNA interactome and proteins regulated upon SARS-CoV-2 infection in Huh7 cells. Statistical test: Fisher’s exact test with Benjamini–Hochberg adjustment.

Supplementary Table 9 CNBP eCLIP peaks and target transcript information (human).

Supplementary Table 10 LARP1 eCLIP peaks and target transcript information (human).

Supplementary Table 11 Gene Ontology enrichment analysis for LARP1 target transcripts (human). Statistical test: Fisher’s exact test with Benjamini–Hochberg adjustment.

## Data Availability

The original mass spectra for all experiments and the protein sequence databases used for the searches have been deposited with the MassIVE repository (https://massive.ucsd.edu) and can be accessed at ftp://massive.ucsd.edu/MSV000085734/. The high-throughput sequencing data have been deposited with the Gene Expression Omnibus under the accession no. GSE154430. Source data are provided with this paper.

## Source data

Source Data Fig. 4 Unprocessed western blots for Fig. 4. Source Data Extended Data Fig. 1 Unprocessed western blots for Extended Data Fig. 1. Source Data Extended Data Fig. 4 Unprocessed western blots for Extended Data Fig. 4.

## Code availability

The computer code for the custom analyses is publicly available at https://munschauerlab.github.io/SCoV2-proteome-atlas/.
