# Supplementary material for: The SARS-CoV-2 RNA–protein interactome in infected human cells
Source: Nat Microbiol. 2020 Dec 21;6(3):339–53. doi: 10.1038/s41564-020-00846-z (PMC7906908; doi:10.1038/s41564-020-00846-z)
Supplement: Supplementary file 1 — Reporting Summary [file 41564_2020_846_MOESM1_ESM.pdf]

nature research

Corresponding author(s): Jochen Bodem, Mathias Munschauer

Last updated by author(s): Nov 27, 2020

Reporting Summary

Nature Research wishes to improve the reproducibility of the work that we publish. This form provides structure for consistency and transparency in reporting. For further information on Nature Research policies, see our Editorial Policies and the Editorial Policy Checklist.

Statistics

For all statistical analyses, confirm that the following items are present in the figure legend, table legend, main text, or Methods section.

n/a	Confirmed	
☐	☒	The exact sample size (*n*) for each experimental group/condition, given as a discrete number and unit of measurement
☐	☒	A statement on whether measurements were taken from distinct samples or whether the same sample was measured repeatedly
☐	☒	The statistical test(s) used AND whether they are one- or two-sided<br>*Only common tests should be described solely by name; describe more complex techniques in the Methods section.*
☒	☐	A description of all covariates tested
☐	☒	A description of any assumptions or corrections, such as tests of normality and adjustment for multiple comparisons
☐	☒	A full description of the statistical parameters including central tendency (e.g. means) or other basic estimates (e.g. regression coefficient) AND variation (e.g. standard deviation) or associated estimates of uncertainty (e.g. confidence intervals)
☐	☒	For null hypothesis testing, the test statistic (e.g. *F*, *t*, *r*) with confidence intervals, effect sizes, degrees of freedom and *P* value noted<br>*Give P values as exact values whenever suitable.*
☒	☐	For Bayesian analysis, information on the choice of priors and Markov chain Monte Carlo settings
☒	☐	For hierarchical and complex designs, identification of the appropriate level for tests and full reporting of outcomes
☒	☐	Estimates of effect sizes (e.g. Cohen's *d*, Pearson's *r*), indicating how they were calculated

Our web collection on statistics for biologists contains articles on many of the points above.

Software and code

Policy information about availability of computer code

Data collection	Spectrum Mill MS Proteomics Workbench, Illumina NextSeq 500 system and software
Data analysis	Graphpad Prism 9.0, Microsoft Excel(v.16.16.27), R (v.4.0.2), Python (v2.7; v3.8), MACS2 (v2.2.7), PicardTools (v2.22.0) bwa (v0.7.17), clusterProfiler (v3.18), MEME Suite (v5.2), deepTools (v2.0), Integrative Genomics Viewer (v2.6.0), Database for Annotation Visualization and Integrated Discovery (DAVID v6.8), MSigDB (v7.2) database, STRING (v11) database, The Drug Gene Interaction Database (DGIdb) (v3.0).<br><br>Computer code for custom analyses is publicly available at: https://munschauerlab.github.io/SCoV2-proteome-atlas/.

For manuscripts utilizing custom algorithms or software that are central to the research but not yet described in published literature, software must be made available to editors and reviewers. We strongly encourage code deposition in a community repository (e.g. GitHub). See the Nature Research guidelines for submitting code & software for further information.

Data

Policy information about availability of data

All manuscripts must include a data availability statement. This statement should provide the following information, where applicable:

- Accession codes, unique identifiers, or web links for publicly available datasets
- A list of figures that have associated raw data
- A description of any restrictions on data availability

The original mass spectra for all experiments, and the protein sequence databases used for searches have been deposited in the public proteomics repository MassIVE (https://massive.ucsd.edu) and are accessible at ftp://massive.ucsd.edu/MSV000085734/.

High-throughput sequencing data are in Gene Expression Omnibus (GEO) and are available under the accession number GSE154430.

Field-specific reporting

Please select the one below that is the best fit for your research. If you are not sure, read the appropriate sections before making your selection.

☒ Life sciences ☐ Behavioural & social sciences ☐ Ecological, evolutionary & environmental sciences

For a reference copy of the document with all sections, see nature.com/documents/nr-reporting-summary-flat.pdf

Life sciences study design

All studies must disclose on these points even when the disclosure is negative.

Sample size	No sample size calculations were performed a priori. Sample sizes were determined following best practices in the field. We performed at least 3 independent experiments for all assays unless otherwise noted.
Data exclusions	Mass spectrometry data were filtered for common laboratory contaminants and keratins. Otherwise no data were excluded.
Replication	As reported in the figure legends, main text and Method section, the findings were reliably reproduced.
Randomization	There were no variables or interventions to randomize in this study.
Blinding	Blinding is not relevant to our study, as our tools are not dependent on blinding. Investigators could not be blinded during data collection or analysis. Analyses were performed in an exploratory manner where blinding is not possible.

Reporting for specific materials, systems and methods

We require information from authors about some types of materials, experimental systems and methods used in many studies. Here, indicate whether each material, system or method listed is relevant to your study. If you are not sure if a list item applies to your research, read the appropriate section before selecting a response.

Materials & experimental systems

n/a	Involved in the study	
☐	☒	Antibodies
☐	☒	Eukaryotic cell lines
☒	☐	Palaeontology and archaeology
☒	☐	Animals and other organisms
☒	☐	Human research participants
☒	☐	Clinical data
☒	☐	Dual use research of concern

Methods

n/a	Involved in the study	
☒	☐	ChIP-seq
☒	☐	Flow cytometry
☒	☐	MRI-based neuroimaging

Antibodies

Antibodies used	CNBP - Proteintech #67109-1-Ig (IP, Western Blot)<br>SARS-CoV-2 Nucleoprotein - Abcam #ab272852 (Western blot)<br>POP1 - Proteintech #12029-1-AP (Western blot)<br>LARP1 - Bethyl #A302-087A (IP, Western blot)<br>TUBULIN - Cell Signaling Technologies #2144 (Western blot)<br>ACTIN - Santa Cruz #sc-47778 (Western blot)<br>IRDye 800CW Goat anti-Rabbit IgG - LI-COR #926-32211 (Western blot)<br>IRDye 800CW Goat anti-Mouse IgG - LI-COR #926-32210 (Western blot)
Validation	Antibodies were validated by the manufacturer and relevant data is available at the manufacturer's website.

Eukaryotic cell lines

Policy information about cell lines

Cell line source(s)	Huh7 and Calu3 (provided by the Virology Diagnostics Unit at Institute of Virology and Immunobiology, University of Würzburg), ACE2-A549 (a generous gift from Andreas Pichlmair), Vero-E6-TMPRSS2 cells (a generous gift from Stefan Pöhlmann), HEK293 (provided by the Utz Fischer laboratory).

Authentication	Cell lines were authenticated by the provider.
Mycoplasma contamination	Cell lines regularly tested negative for mycoplasma contamination.
Commonly misidentified lines (See ICLAC register)	No commonly misidentified cell lines were used.
