# Supplementary figures and images for: The SARS-CoV-2 RNA–protein interactome in infected human cells
Source: Nat Microbiol. 2020 Dec 21;6(3):339–53. doi: 10.1038/s41564-020-00846-z (PMC7906908; doi:10.1038/s41564-020-00846-z)

Original images used to generate Figure 4b

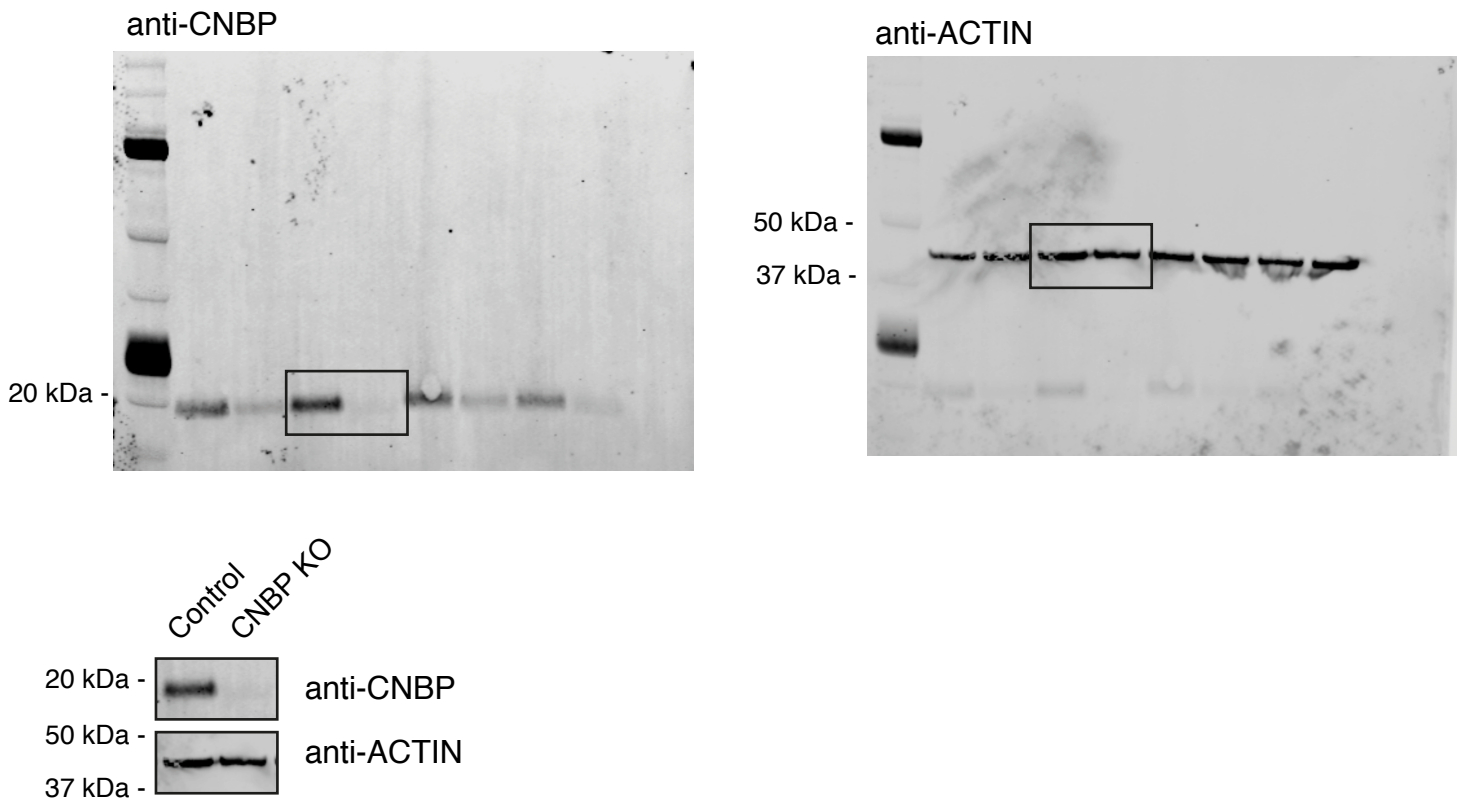

Supplement: Source Data Fig. 4 — Unprocessed western blots for Fig. 4. [file 41564_2020_846_MOESM13_ESM.pdf]

Original images used to generate Extended Data Figure 1e

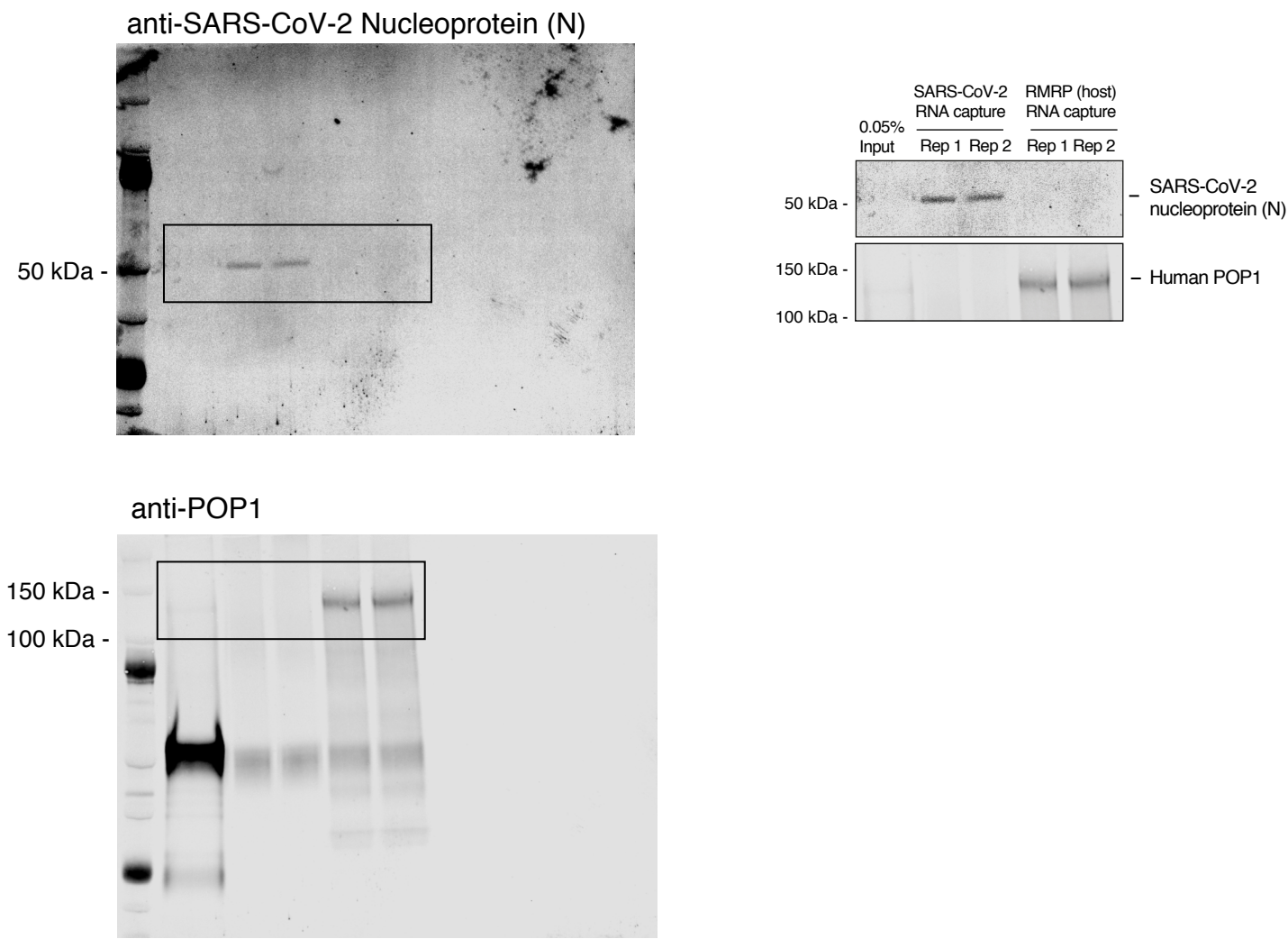

Supplement: Source Data Extended Data Fig. 1 — Unprocessed western blots for Extended Data Fig. 1. [file 41564_2020_846_MOESM14_ESM.pdf]
